# TENET 2.0: Identification of key transcriptional regulators and enhancers in lung adenocarcinoma

**DOI:** 10.1371/journal.pgen.1009023

**Published:** 2020-09-14

**Authors:** Daniel J. Mullen, Chunli Yan, Diane S. Kang, Beiyun Zhou, Zea Borok, Crystal N. Marconett, Peggy J. Farnham, Ite A. Offringa, Suhn Kyong Rhie

**Affiliations:** 1 Department of Biochemistry and Molecular Medicine and the Norris Comprehensive Cancer Center, Keck School of Medicine, University of Southern California, CA, United States of America; 2 Department of Surgery, Keck School of Medicine, University of Southern California, CA, United States of America; 3 Hastings Center for Pulmonary Research and Division of Pulmonary, Critical Care and Sleep Medicine, Department of Medicine, Keck School of Medicine, University of Southern California, CA, United States of America; St Jude Children's Research Hospital, UNITED STATES

## Abstract

Lung cancer is the leading cause of cancer-related death and lung adenocarcinoma is its most common subtype. Although genetic alterations have been identified as drivers in subsets of lung adenocarcinoma, they do not fully explain tumor development. Epigenetic alterations have been implicated in the pathogenesis of tumors. To identify epigenetic alterations driving lung adenocarcinoma, we used an improved version of the Tracing Enhancer Networks using Epigenetic Traits method (TENET 2.0) in primary normal lung and lung adenocarcinoma cells. We found over 32,000 enhancers that appear differentially activated between normal lung and lung adenocarcinoma. Among the identified transcriptional regulators inactivated in lung adenocarcinoma *vs*. normal lung, NKX2-1 was linked to a large number of silenced enhancers. Among the activated transcriptional regulators identified, CENPA, FOXM1, and MYBL2 were linked to numerous cancer-specific enhancers. High expression of CENPA, FOXM1, and MYBL2 is particularly observed in a subgroup of lung adenocarcinomas and is associated with poor patient survival. Notably, CENPA, FOXM1, and MYBL2 are also key regulators of cancer-specific enhancers in breast adenocarcinoma of the basal subtype, but they are associated with distinct sets of activated enhancers. We identified individual lung adenocarcinoma enhancers linked to CENPA, FOXM1, or MYBL2 that were associated with poor patient survival. Knockdown experiments of FOXM1 and MYBL2 suggest that these factors regulate genes involved in controlling cell cycle progression and cell division. For example, we found that expression of *TK1*, a potential target gene of a MYBL2-linked enhancer, is associated with poor patient survival. Identification and characterization of key transcriptional regulators and associated enhancers in lung adenocarcinoma provides important insights into the deregulation of lung adenocarcinoma epigenomes, highlighting novel potential targets for clinical intervention.

## Author summary

Although genetic alterations have been identified as drivers in subsets of lung adenocarcinoma, they do not fully explain tumor development. Here we investigated epigenetic alterations that might contribute to lung adenocarcinoma progression. We used a bioinformatics approach called TENET 2.0 to identify key regulators and enhancers altered in lung adenocarcinoma compared to normal lung. We identified NKX2-1 as a transcriptional regulator inactivated in lung adenocarcinoma, linked to a large number of enhancers silenced in cancer. Among the activated transcriptional regulators, CENPA, MYBL2, and FOXM1 were linked to numerous cancer-specific enhancers and high expression of these regulators was observed in a subgroup of lung adenocarcinomas showing poor patient survival. Investigating downstream effects of these key regulators, we also determined individual enhancers associated with survival and their potential target genes, including *TK1*. Our findings suggest that abnormal expression of key regulators drives epigenetic deregulation in lung adenocarcinoma, promoting future development of novel biomarkers and therapeutic targets.

## Introduction

Lung cancer is the second most commonly diagnosed form of cancer and the leading cause of cancer-related death in both men and women [1]. Lung adenocarcinoma (LUAD) arises in the alveolar epithelium of the lung and comprises almost 50% of all lung cancer cases in the United States [2]. Major risk factors for LUAD include tobacco smoking, inherited genetic factors, diet, alcohol consumption, exposure to sources of ionizing radiation and environmental contaminants [3,4]. These risk factors induce molecular and cellular changes in alveolar epithelial cells, leading these purported cells of origin to form LUAD. A number of somatic genetic alterations such as *KRAS*, *EGFR*, *NF1*, and *BRAF* mutations, gene fusions involving *ALK*, *EML4*, and *ROS1*, and copy number variations of the *KRAS* and *EGFR* genes have been identified and utilized in the development of targeted therapies for LUAD [5,6]. However, approximately a quarter of LUAD cases do not possess any of these genetic alterations [7], suggesting that other molecular changes likely contribute to lung cancer development.

Epigenomic features do not affect the sequence of DNA, but can affect the transcriptional output of genes in a cell-type specific manner by altering the activity of regulatory elements such as promoters (which are located proximal to the transcription start site of genes) and enhancers (which can be found at a great genomic distance (distal) from their target genes). Promoters and enhancers play critical roles in ensuring cell type specificity by controlling gene expression through the binding of transcription factors and recruitment of the transcriptional machinery [8,9]. Disruption of epigenetic marks and altered activity of regulatory elements may lead to the development of cancer [10,11]. The epigenetic state at regulatory elements can be determined by measuring levels of histone 3 lysine 4 trimethylation (H3K4me3, a promoter mark) or histone 3 lysine 27 acetylation (H3K27ac, an enhancer mark), using chromatin immunoprecipitation (ChIP)-seq. Epigenetic states can also be assessed by using open chromatin assays such as DNase-seq, NOMe-seq (Nucleosome Occupancy and Methylome), or ATAC-seq (assay for transposase-accessible chromatin) [12]. DNA methylation levels can be used to infer accessibility of open chromatin regions at regulatory elements since active promoters and enhancers tend to be unmethylated [13,14]. Moreover, the binding of activated transcription factors affects DNA methylation states of targeted regulatory elements [13–17].

We previously developed the Tracing Enhancer Networks using Epigenetic Traits (TENET) method which can identify differentially activated enhancers and their associated transcriptional regulators (TRs) using tumor *vs*. normal tissue samples. TENET incorporates information from ChIP-seq and open chromatin assays to determine the location of enhancers in normal and tumor tissues, and uses the DNA methylation levels of probes in the identified regions as an indicator of enhancer activity. Then, TENET uses gene expression data from the same samples to identify transcriptional regulators whose expression levels are highly correlated with the DNA methylation level of each enhancer. Using TENET, biomarkers and potential oncogenic drivers of breast cancer (e.g. GATA3, ESR1, FOXA1), prostate cancer (e.g. FOXA1, HOXC6, HOXB13), and kidney cancer (e.g. GLIS1, MAF, RUNX1) have been identified [14]. These results illustrate the utility of the TENET method to identify key transcriptional regulators associated with tumorigenesis. However, computational time and power associated with identification of the key transcriptional regulators of the original TENET method was not optimal. Here, we have significantly improved the method, updating the databases, including new algorithms to identify epigenetic traits associated with mortality, and greatly decreasing the computational time needed. We then applied the improved version of the method (TENET 2.0) to numerous epigenome and transcriptome datasets from lung and lung cancer and identified key transcriptional regulators and enhancers associated with lung adenocarcinoma, providing novel potential targets for clinical intervention.

## Results

### Identification of differentially activated enhancers in normal lung *versus* lung adenocarcinoma

**Fig 1:**
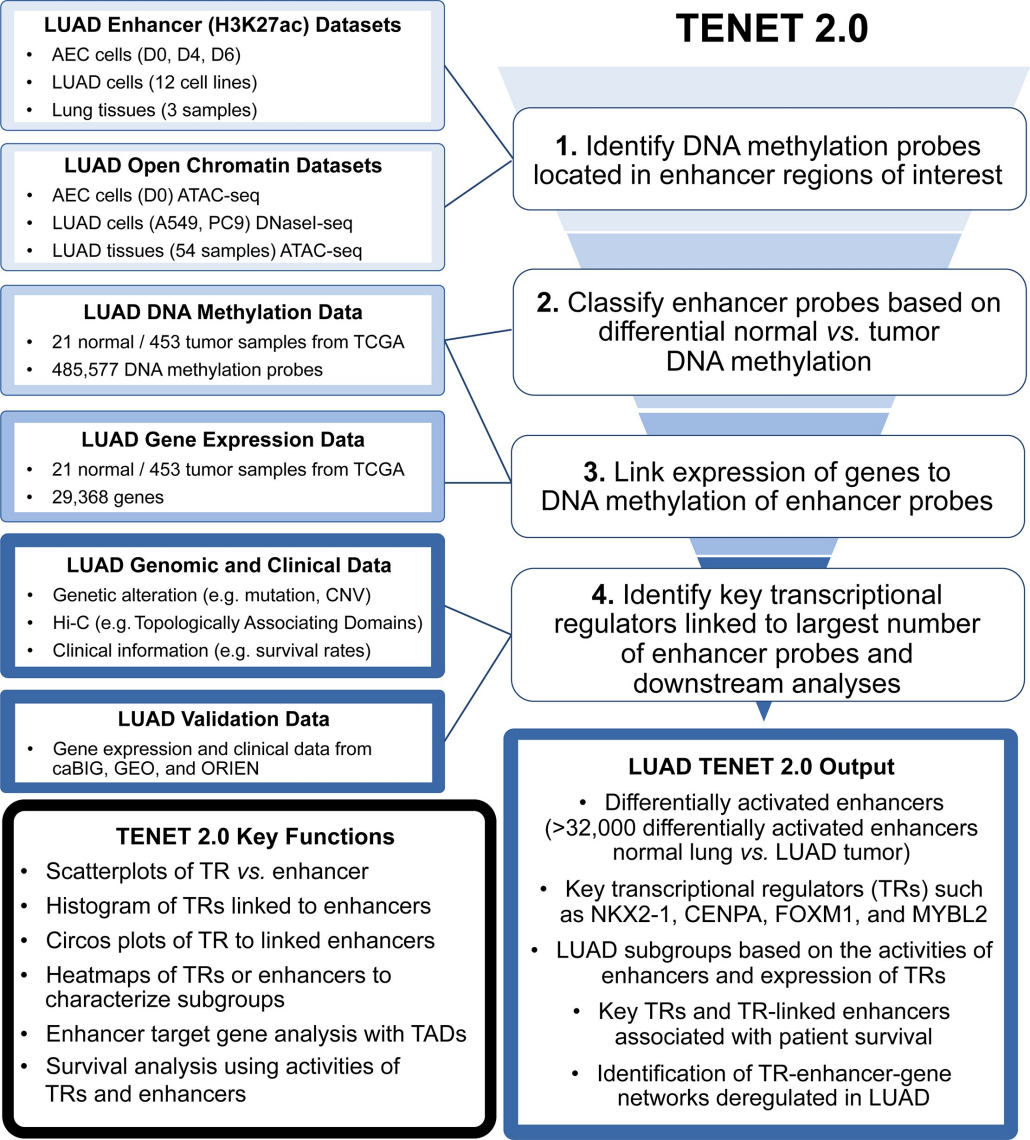
A workflow of TENET 2.0. First, DNA methylation probes marking enhancer regions of interest are identified by overlapping them with both H3K27ac ChIP-seq datasets and open chromatin regions. Next, enhancer probes are classified based on their DNA methylation level in the tumor *vs*. normal samples and linked to the expression of genes to identify key transcriptional regulators (TRs). Using genetic alteration, Hi-C topologically associating domain (TAD), and clinical information, identified key TRs and TR-enhancer-gene networks are characterized. Additional gene expression and clinical data are used to validate findings of key TRs. Lung-related datasets used for this study are shown at left. The output from this LUAD study is indicated in the middle bottom box. The left bottom box summarizes key TENET 2.0 functions.

Each cell type has a distinct transcriptome, which is established by the levels and activities of transcriptional regulators that bind to regulatory elements and control the expression of numerous target genes. Among regulatory elements, the activity of enhancers is most closely linked to cell identity, as they are often bound by cell-type specific transcriptional regulators [18]. We developed TENET 2.0 to identify key transcriptional regulators whose expression levels are associated with changes in DNA methylation levels at enhancers in normal *vs*. tumor tissue samples (Fig 1 and S1 Fig). TENET 2.0 now utilizes human reference genome hg38 and includes updated databases of human genes (GENCODE v22) [19]. To comprehensively characterize and identify transcriptional regulators altered in tumors, we used the transcription factor database specified by Lambert et al. [20]. We developed TENET 2.0 to have increased processing speed, compared to the original version, and have also included new algorithms to assess the relationship with patient survival, among others.

To study transcriptional enhancer networks in LUAD using TENET 2.0, we first identified lung-relevant enhancer regions. Alveolar epithelial cells (AECs) are the presumed cells of origin of LUAD [21]. There are two types of alveolar epithelial cells: cuboidal type 2 cells (AT2), which are involved in surfactant production and serve as facultative progenitors post-injury, and large, delicate type 1 cells (AT1), which cover the majority of the alveolar surface and mediate gas exchange. While AT2 cells are the suspected cells of origin of lung adenocarcinoma, the possible role of AT1 cells has not been well investigated due to the difficulty in manipulating these fragile cells. Thus, we incorporated both populations of these cells into our study. We first purified human AT2 cells and then used an *in vitro* differentiation protocol (which mimics aspects of normal lung re-epithelialization) to derive AT1-like cells [22,23]. We then generated H3K27ac ChIP-seq data from the AT2 cells (day 0), transitional cells (day 4), and differentiated AT1-like cells (day 6). We also used H3K27ac ChIP-seq data from normal lung tissue samples and LUAD cells downloaded from the Roadmap Epigenomics Project (REMC) [24], the Encyclopedia of DNA Elements Project (ENCODE) [25,26], and the DataBase of Transcriptional Start Sites (DBTSS) [27]. Because tumorigenesis might activate enhancers that are not normally active in the lung, we also included H3K27ac ChIP-seq from 98 different cell types collected from REMC [24] and ENCODE [25,26]. We next delineated the open chromatin regions where the transcription factors bind within each enhancer, using ATAC-seq peaks generated in-house from AECs, ATAC-seq peaks from LUAD tissues and cell lines downloaded from other studies [28–30], DNaseI hypersensitive sites from LUAD cell lines, and a collected list of DNaseI hypersensitive sites from 125 different tissues and cell lines from ENCODE [25,26]. A list of datasets we used for this study can be found in S1 Table, and identified enhancer and open chromatin regions can be found in S2 Table. Finally, DNA methylation probes from the Illumina Infinium Human Methylation 450K (HM450) array that are contained within the open chromatin region of each enhancer were selected. As enhancers are bound by cell-type specific transcription factors and more cell-type and individual specific than promoters [12], we focused on enhancers for our analyses using only probes located >1.5 kb from transcription start sites. In all, we identified 76,765 "enhancer probes" that can be studied using lung tissue samples (S3A Table); on average, one probe was found per open chromatin region in each enhancer.

**Fig 2:**
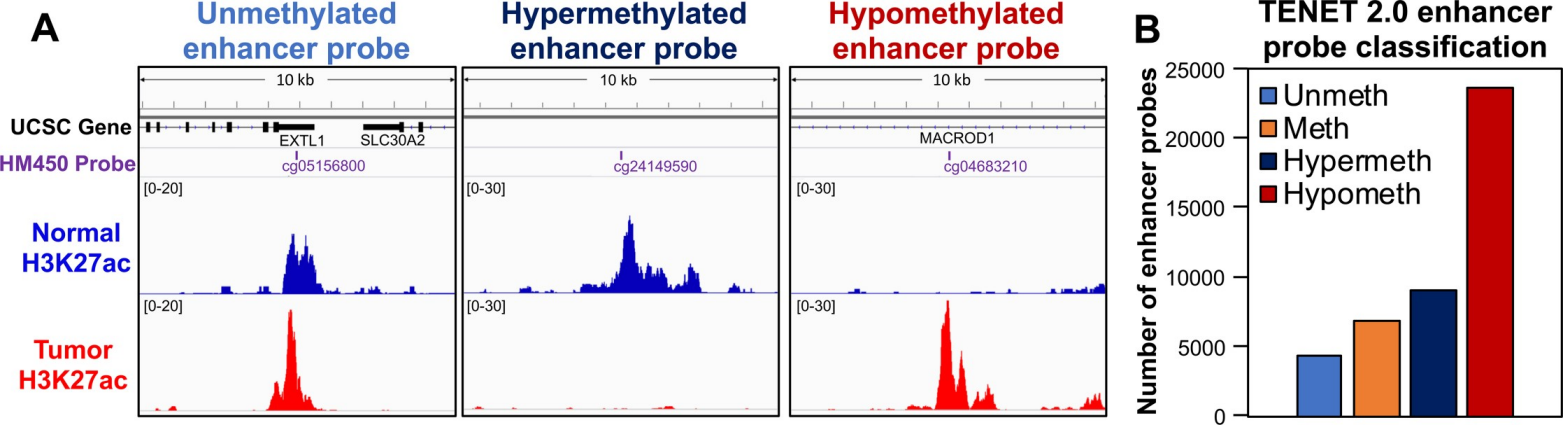
**Identification of differentially-methylated enhancer probes** (A) Integrative Genomics Viewer (IGV) screenshots show 10 kb of the genomic context centered on example probes, with UCSC gene annotations (GENCODE v22) in the vicinity, the name and location of the probe, and the H3K27ac signal from AEC (normal) as well as A549 cells (LUAD cell line). The unmethylated probe shows an active enhancer region in both the AEC and A549 cells. The hypermethylated probe shows an active enhancer region found in only the AEC, indicating an enhancer that is inactive in tumors, while the hypomethylated probe displays marks in only A549 cells, indicating an enhancer that is activated in tumors. (B) Categorization of the identified enhancer probes by activity.

Having collected the above information, we next assessed the differential activities of all of the enhancers in normal lung *vs*. LUAD tumor samples. For this, we collected DNA methylation data for the enhancer probes (n = 76,765) from 453 LUAD tissue samples and 21 histologically normal lung tissue samples adjacent to tumors from The Cancer Genome Atlas (TCGA) [7] (S4 Table). By comparing the DNA methylation level (as a reflection of enhancer activity) of each probe in the normal *vs*. tumor samples, we classified the enhancer probes into 4 groups: methylated (“constitutively inactive”), i.e. highly methylated in both normal and tumor samples; unmethylated (“constitutively active”), i.e. lowly methylated in both normal and tumor samples; hypermethylated (“normal-specific”; inactivated in LUAD), i.e. showing low methylation in normal samples but higher methylation in tumor samples; and hypomethylated (“cancer-specific”; activated in LUAD), i.e. showing high methylation in normal samples, but lower methylation in tumor samples. For example, the unmethylated probe cg05156800, located in an enhancer region on chr1p36.11 near the 3'UTR of *EXTL1*, marks an enhancer that is active in both normal lung and LUAD tumors (Fig 2A, left panel). In contrast, the hypermethylated probe cg24149590 in an intergenic region on chr14q24.3 is located in an enhancer, active in normal lung but not in LUAD (Fig 2A, middle panel). Hypomethylated probe cg04683210, located in an intron of *MACROD1* on chr11q13.1 marks an enhancer that is active LUAD but not in normal AECs (Fig 2A, right panel). Using this classification scheme, we identified 4,344 unmethylated, 6,830 methylated, 9,056 hypermethylated, and 23,583 hypomethylated enhancer probes. An excess of identified hypomethylated probes suggests that enhancer activation is a common molecular alteration in LUAD (Fig 2B, S3A and S3B Table).

### Identification of key transcriptional regulators dysregulated in lung adenocarcinoma

**Fig 3:**
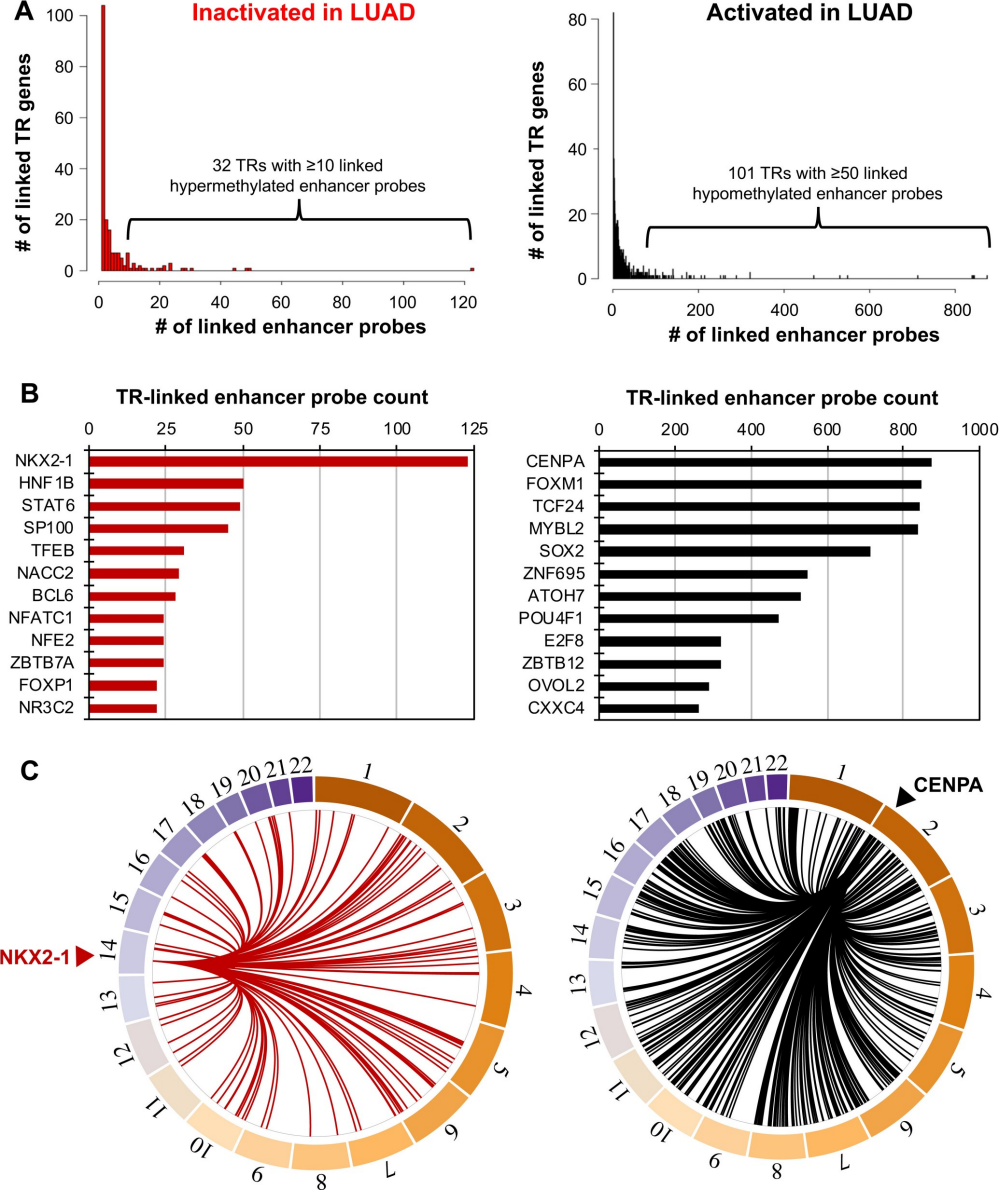
**Identification of key dysregulated transcriptional regulators in LUAD** (A) The left histogram shows the number of inactivated (hypermethylated) enhancer probes per inactivated transcriptional regulator (TR), and the right shows the number of activated (hypomethylated) enhancer probes per activated TR. Most TRs were linked to relatively few enhancer probes. However, 31 inactivated TRs in LUAD were linked to 10 or more hypermethylated enhancer probes, and 101 activated TRs in LUAD were linked to 50 or more hypomethylated enhancer probes. (B) Number of enhancer links for top 12 transcriptional regulators. Inactivated TRs are shown at left, while activated TRs are shown at right. (C) Circos plots show the link between the top inactivated TR (left, NKX2-1) and activated TR (CENPA, right) and their associated enhancers throughout the genome.

Having identified over 32,000 differentially activated enhancer probes between normal lung and LUAD, we next used matched gene expression data to test the association between the expression of each known human transcriptional regulator (n = 1,639) and the level of DNA methylation (as a measure of accessibility and thus activity) of each enhancer probe, using TENET 2.0. We identified 1) inactivated transcriptional regulators that showed a correlation of lower expression with increased DNA methylation of enhancer probes in a subset of LUAD samples (candidate tumor suppressors), and 2) activated transcriptional regulators that showed a correlation of higher expression with decreased DNA methylation of enhancer probes in a subset of LUAD samples (candidate oncogenes) (S1 Fig). Most of the known 1,639 human transcriptional regulators we interrogated were linked to relatively few cell-type specific enhancer probes (Fig 3A). However, a subset of transcriptional regulators was found to be linked to many cell-type specific enhancer probes (Fig 3A).

We found that 31 inactivated transcriptional regulators were found to be linked to 10 or more hypermethylated enhancer probes (S5A Table). For example, NKX2-1 and HNF1B were linked to 123 and 50 hypermethylated enhancer probes, respectively (Fig 3B and 3C, S5A Table). NKX2-1, the top transcriptional regulator inactivated in LUAD, linked to the largest number of enhancers silenced in LUAD, is known to play an important role in lung development and maintenance of AEC cell identity [31]. NKX2-1 also acts as an activator of HOP (Hsp70/Hsp90 Organizing Protein), a potential tumor suppressor gene in lung cancer, inhibiting epithelial to mesenchymal transition [32]. HNF1B is previously reported to act as a tumor suppressor in several tumors, including renal cancer, ovarian cancer, and prostate cancer [33–35]. Our finding that lower expression of HNF1B is linked to many inactivated enhancers in LUAD suggests that it may also act as a tumor suppressor in lung cancer.

**Fig 4:**
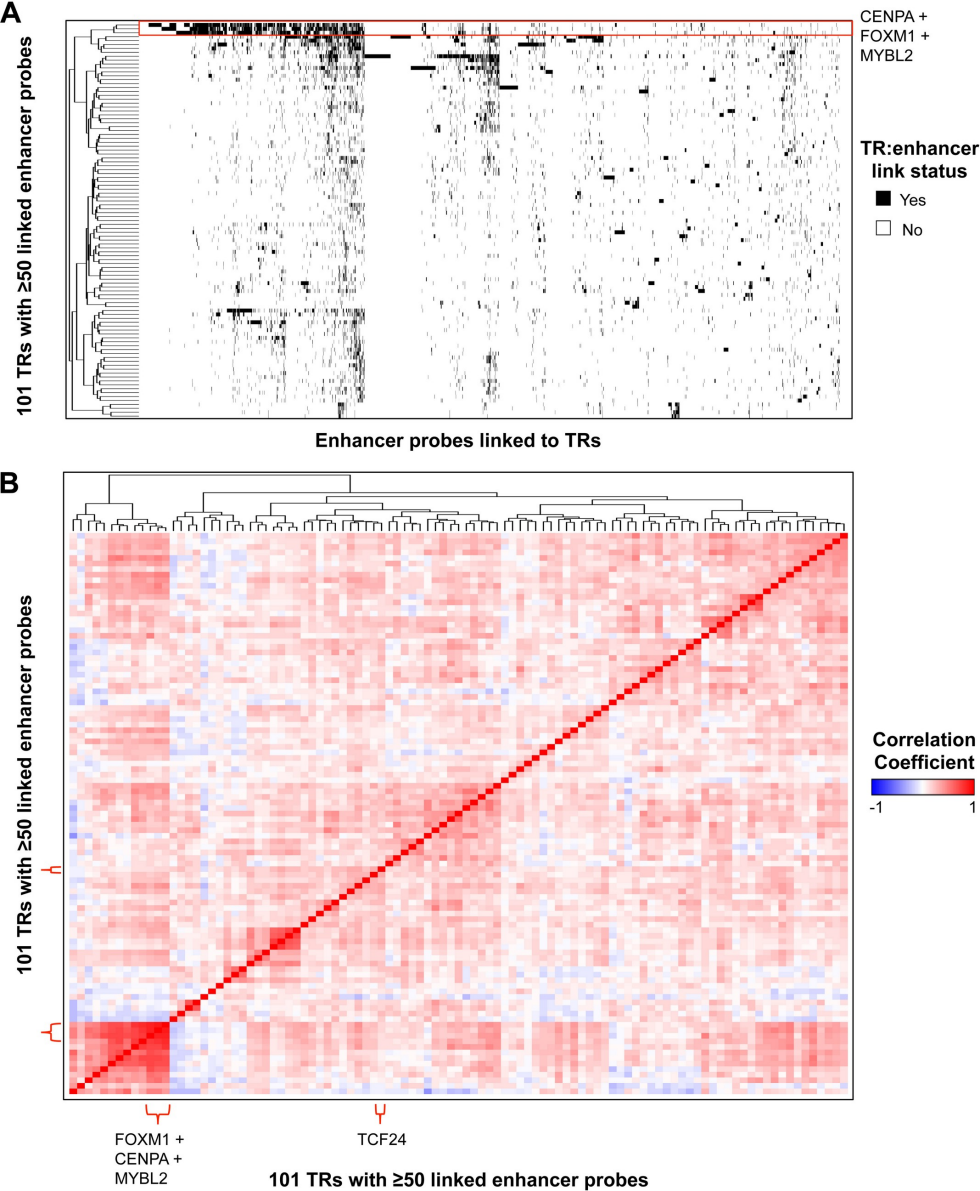
**Interaction of key transcriptional regulators activated in LUAD** (A) Interaction map of the top 101 transcriptional regulators and the 3,682 total unique hypomethylated probes linked to those genes. CENPA, FOXM1, and MYBL2 show strong overlap in linked probes (red box). (B) Heatmap of pairwise expression correlation values between each of the top 101 transcriptional regulators. FOXM1, CENPA, and MYBL2 show a high degree of correlation with each other (r^2^>0.7), but TCF24 (one of the top 4 most highly linked TRs; Fig 3B) does not (r^2^<0.1).

On the other hand, we found 101 activated transcriptional regulators linked to 50 or more hypomethylated probes (S5C Table). The top activated transcriptional regulators were CENPA, FOXM1, TCF24, and MYBL2, which were linked to 875, 845, 843, and 840 cancer-specific enhancer probes, respectively (Fig 3B and 3C, S5C Table). These transcriptional regulators likely have the largest influence on the transcriptomes of lung adenocarcinoma tumors by changing the activities of many enhancers. Therefore, we further investigated the identified activated transcriptional regulators associated with many cancer-specific activated enhancers. To determine whether these transcriptional regulators control the activity of distinct enhancers or cooperate with each other to regulate the same set of enhancers, we generated an interaction map displaying the association of the 3,682 cancer-specific enhancer probes linked to at least one of the 101 transcriptional regulators (Fig 4A). Interestingly, CENPA, FOXM1, and MYBL2 showed considerable overlap in their sets of linked probes; over 75% of each of their linked probes was also linked to a probe in the set of at least one of the other two transcriptional regulators (Fig 4A—red box, S5E Table). The overlap between these transcriptional regulators is much higher than with other key transcriptional regulators identified (e.g. TCF24, SOX2). Examination of the expression levels of each of the 101 top-ranked transcriptional regulators showed that the expression levels of *CENPA*, *FOXM1*, and *MYBL2* were highly correlated with each other (r^2^>0.7) across all profiled LUAD samples (Fig 4B—red brackets, S5F Table). We validated these results using an additional transcriptomic dataset obtained from other lung tumor tissue samples from ORIEN (Oncology Research Information Exchange Network) (www.oriencancer.org) (S2 Fig). These results suggest that these 3 transcriptional regulators may work together to activate a common set of cancer-specific enhancers.

### Identification of transcriptional regulators whose expression is associated with poor patient survival

**Fig 5:**
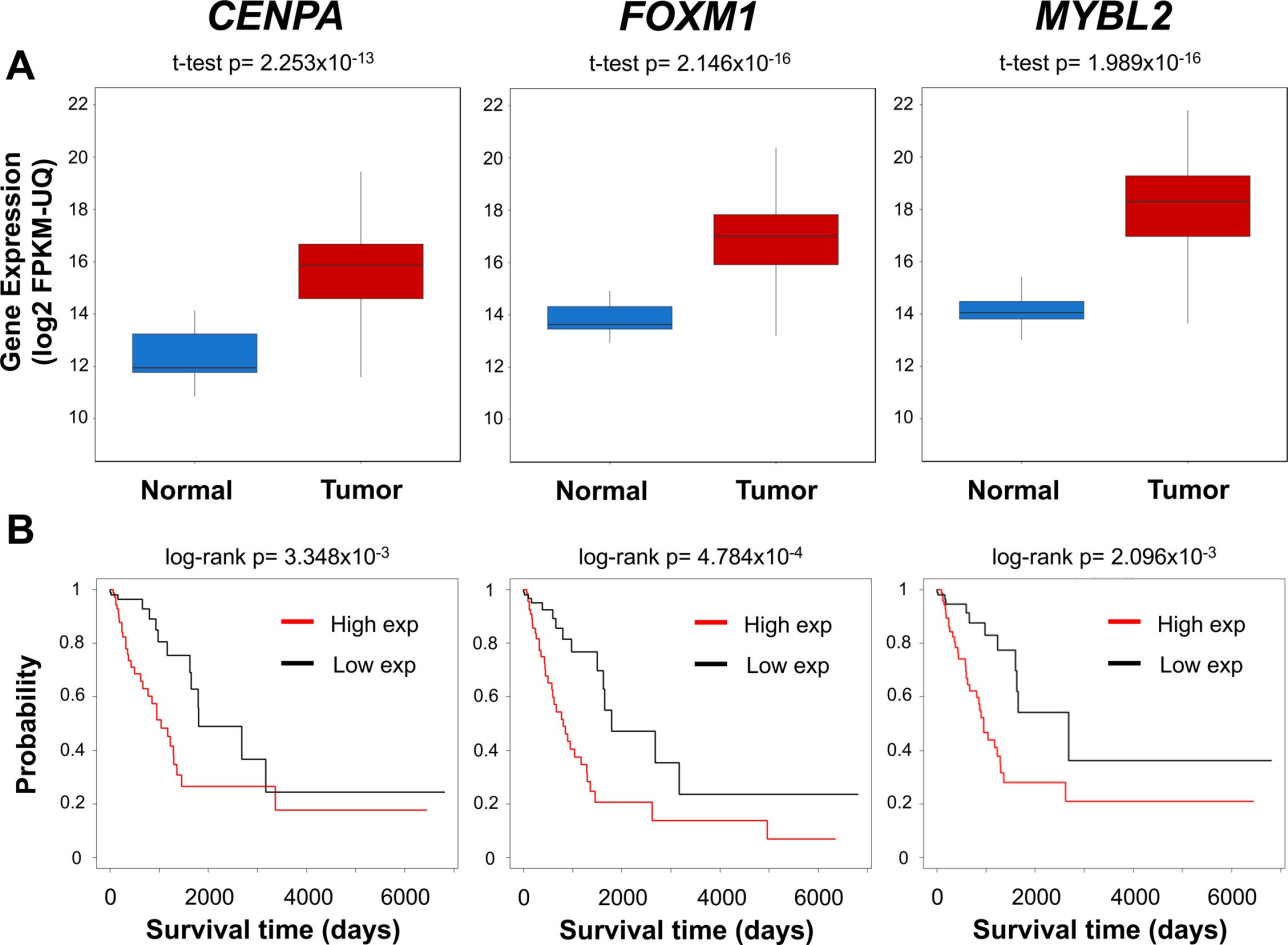
*CENPA*, *FOXM1* and *MYBL2* are highly expressed in tumors and associated with poor patient survival. (A) Boxplots of expression of *CENPA*, *FOXM1* and *MYBL2* in 453 TCGA LUAD and 21 adjacent normal samples. All three genes were significantly upregulated in LUAD. (B) Kaplan-Meier survival plots comparing differences in survival between samples with the highest and lowest quartiles of *CENPA*, *FOXM1* and *MYBL2* expression. Survival was compared using TCGA LUAD samples.

To further investigate the role of key transcriptional regulators activated in LUAD, we more closely examined gene expression levels in normal *vs*. tumor tissues. Of the top 12 transcriptional regulators, *CENPA*, *FOXM1*, and *MYBL2* were among the most highly expressed and displayed the largest differences in expression between tumor and normal tissues; each was >8 times more highly expressed in LUAD as compared to normal lung (Fig 5A, S3 Fig). Next, we examined the association of transcriptional regulator expression with patient survival, and we found that high expression levels of *CENPA*, *FOXM1*, and *MYBL2* were the most significantly associated with poor patient survival in the TCGA LUAD cohort (Fig 5B, S4 Fig). We validated these results for *CENPA*, *MYBL2*, and *FOXM1* using an additional survival dataset obtained from other LUAD samples [36] (S5 Fig). Expression of *CENPA*, *FOXM1*, and *MYBL2* did not appear to be very strongly associated with age, sex, or cancer stage. However, we found that history of tobacco exposure was correlated with the gene expression of each of the three transcriptional regulators (S6A Fig, S6 Table). Additionally, we found that high total mutation burden was similarly associated with increased expression of these genes in the LUAD tumor samples (S6B Fig).

### CENPA, FOXM1, and MYBL2 are activated in a subgroup of lung adenocarcinoma and breast adenocarcinoma

**Fig 6:**
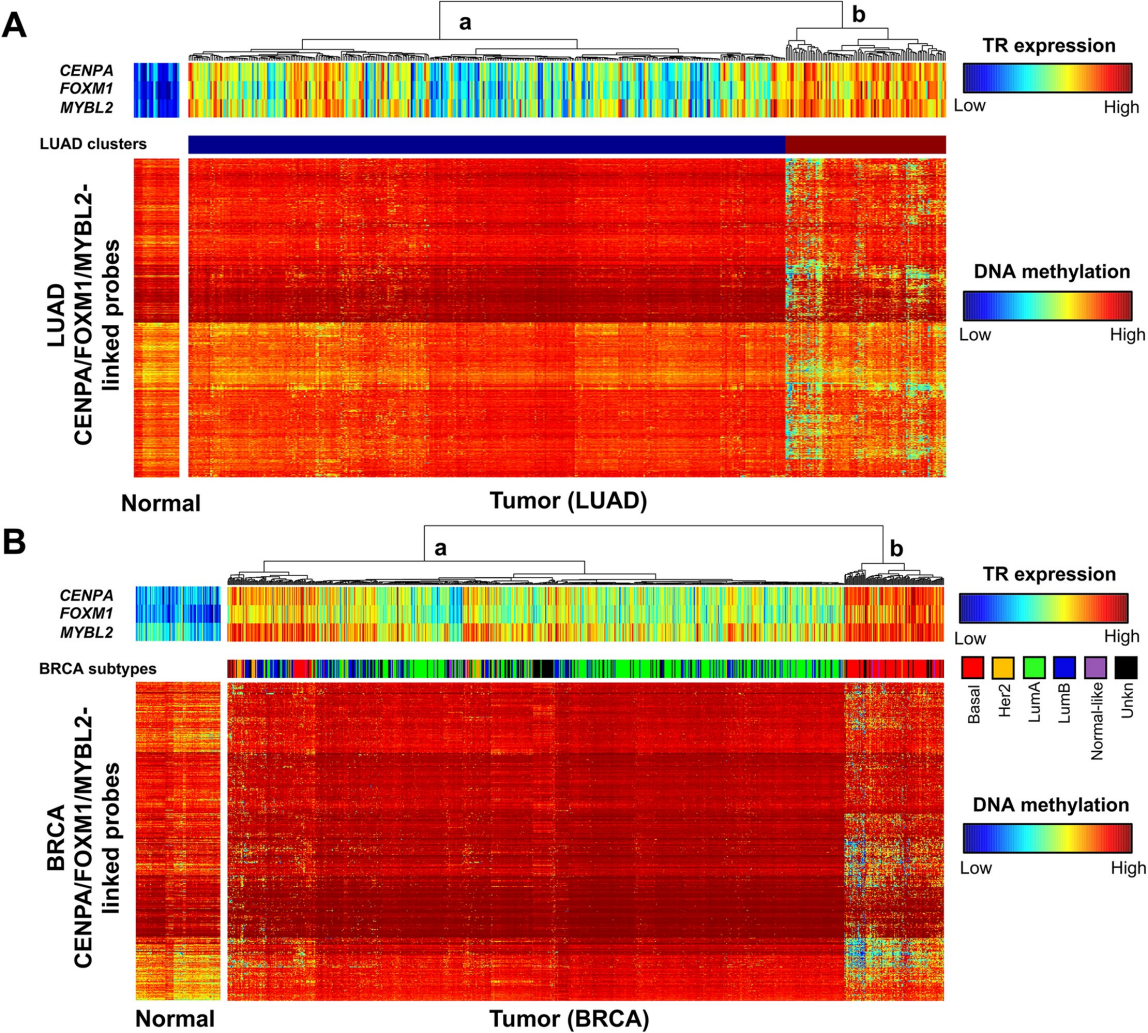
LUAD and BRCA subgroups with activated CENPA, FOXM1 and MYBL2-linked enhancers. (A) DNA methylation heatmap showing *CENPA*, *FOXM1*, and *MYBL2* expression-linked LUAD-specific enhancer probes for normal and LUAD tissue samples. Clusters represent the largest two divisions in LUAD tumor samples as determined by unsupervised clustering. LUAD tumor samples in cluster b display generally higher expression of the 3 transcriptional regulators and broad hypomethylation of CENPA/FOXM1/MYBL2-linked probes. (B) DNA methylation heatmap showing CENPA, FOXM1, and MYBL2-linked breast cancer-specific enhancer probes for normal and BRCA tissue samples. BRCA PAM50 (Prediction Analysis of Microarray 50) subtypes are indicated in the middle bar. Of note is the cluster of samples on the right, comprised predominantly of BRCA tumor samples of the basal subtype, with relatively high expression of the three transcriptional regulators and broad hypomethylation of CENPA/FOXM1/MYBL2-linked probes, similar to what is seen in the subgroup of LUAD samples.

Tumor samples with higher expression of *CENPA*, *FOXM1*, and *MYBL2* appear to harbor relatively more cancer-specific enhancers, suggesting that tumors highly expressing *CENPA*, *FOXM1*, and *MYBL2* may have distinct enhancer profiles (S7A Fig). To investigate this, we generated a DNA methylation heatmap of the enhancer probes linked to these three transcriptional regulators (Fig 6A, S3B Table). We observed a subgroup consisting of LUAD samples that are broadly hypomethylated across these enhancers, and that possess relatively high expression of these three transcriptional regulators together (Fig 6A—cluster b, S8A Fig). These samples did not appear to be associated with age, sex, cancer stage, purity, or cancer stage, but they were slightly associated with smoking history in the TCGA dataset, especially current smoking, as well as total mutational burden (S8A–S8C Fig). We saw no apparent association between genetic alterations to *KRAS*, *EGFR*, *NF1*, *or BRAF* and activation of specifically *CENPA*, *FOXM1*, and *MYBL2*-linked enhancers (S8A Fig). It has been previously shown that activation of KRAS signaling increases expression of *FOXM1* [37], and that *MYBL2* can be regulated by EGFR [38]. We therefore examined the total number of cancer-specific enhancer links in samples with and without *KRAS* or *EGFR* genetic alterations and in the highest quartile and remaining quartiles of expression of *FOXM1* and *MYBL2*, respectively. Samples with the highest quartile of *FOXM1* and *MYBL2* expression possessed a significantly greater number of cancer-specific enhancer links, however, *KRAS* or *EGFR* genetic alteration status was not associated with a significant difference in the number of these links regardless of the *FOXM1* and *MYBL2* expression level (S7B Fig). We also observed that a subgroup of LUAD samples, representing those in the top 10% by number of links to CENPA, FOXM1, and MYBL2 showed poorer survival outcomes than samples which did not possess a link (S8D Fig).

In previous analyses, we found that FOXM1 and MYBL2 were activated in breast adenocarcinoma (BRCA) [14]. Having now identified these as key regulators in LUAD, we sought to determine if different enhancers are linked to FOXM1 and MYBL2 in the two cancer types. We reanalyzed the BRCA data using TENET 2.0 (S3C and S3D Table, S4 Table), and found that CENPA, FOXM1, and MYBL2 were among the top transcriptional regulators in BRCA when ranked by number of linked probes (S9A Fig). However, only a subset of TENET-identified CENPA, FOXM1, and MYBL2-linked enhancer probes were shared between both datasets (S9B Fig). This suggests that although some cancer-specific enhancers are common to LUAD and BRCA (S9C Fig, S7 Table), the enhancers regulated by CENPA, FOXM1, and MYBL2 are largely different between tumor types. To further characterize the BRCA enhancers linked to CENPA, MYBL2, and FOXM1, we generated heatmaps of DNA methylation for enhancer probes linked to any of the three transcriptional regulators in BRCA. Interestingly, we found that BRCA tumor samples that belong to the basal subtype have higher expression of these three transcriptional regulators as well as a larger number of hypomethylated CENPA, FOXM1, and MYBL2-linked enhancers than other BRCA subtypes (i.e. luminal A, luminal B, Her2, normal-like) (Fig 6B).

### Identification of CENPA/FOXM1/MYBL2-linked enhancers associated with poor patient survival and their potential target genes

**Fig 7:**
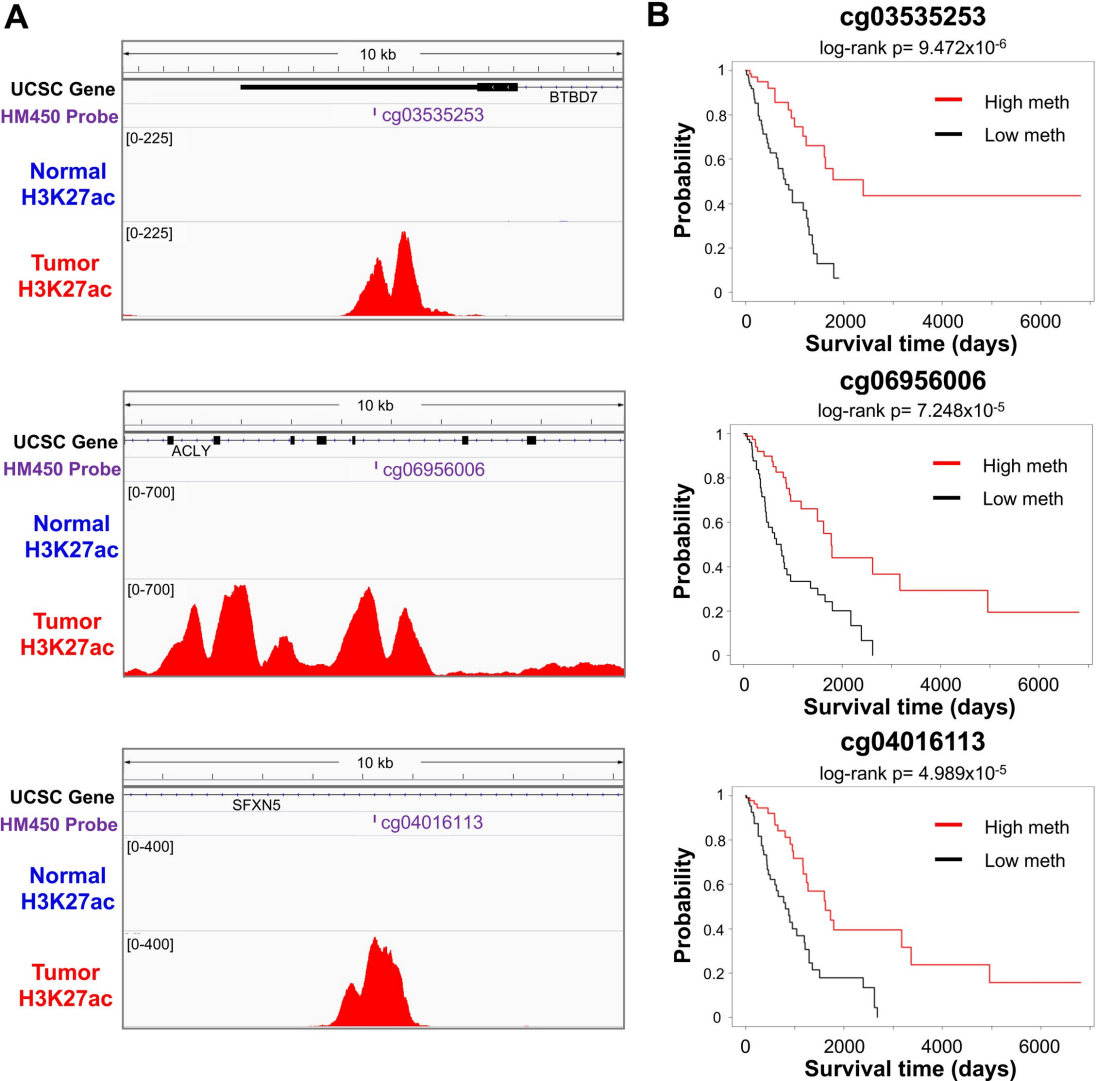
Examples of CENPA/FOXM1/MYBL2-linked enhancer probes associated with survival rate. (A) Shown are three examples of lung cancer-specific enhancers linked to CENPA, FOXM1, or MYBL2 in LUAD. IGV screenshots show 10 kb of the genomic context centered on example probes, with GENCODE v22-annotated UCSC genes in the vicinity, the name and location of the probe, and the H3K27ac signal from normal AEC as well as lung tumor A549 cells. These hypomethylated probes show H3K27ac marks in A549 cells, indicating enhancers active in LUAD but not normal lung tissue. (B) Kaplan-Meier survival plots comparing differences in survival between samples with the highest and lowest quartiles of methylation of the enhancer probe.

We next wondered whether high expression of CENPA, FOXM1, and MYBL2 and the presence of more activated enhancers was clinically relevant. Therefore, we examined the subgroup of LUAD samples, which had high expression of the three transcriptional regulators as well as many enhancer links (over 290 cancer-specific CENPA, FOXM1, or MYBL2 enhancer links), called “highly linked” samples for correlations to overall patient survival (S8A Fig). These "highly linked" samples showed significantly poorer survival outcomes than lowly linked samples (S8D Fig). To further investigate whether any particular cancer-specific enhancers were linked to patient outcome, we performed survival analyses using cancer-specific enhancer probes linked to CENPA, FOXM1, or MYBL2. We found 101 enhancer probes for which lower levels of DNA methylation were associated with poor patient survival (Log-rank p<0.05) (S3B Table). Examples of enhancer probes linked to patient survial included cg03535253, located on chr14q32.12 in the 3'UTR of the *BTBD7* gene, cg06956006, located on chr17q21.2 in an intron of the *ACLY* gene, and cg04016113 in an intron of the *SFXN5* gene on chr2p13.2. Each is located in the vicinity of an active enhancer region in LUAD cells not present in normal AEC, and patients with low levels of methylation of each of these probes (indicating the activation of the enhancer regions) showed significantly poorer survival outcomes (Fig 7).

We next aimed to identify genes and signaling pathways potentially regulated by CENPA, FOXM1, and MYBL2. To this end, we first identified genes within 1 Mb of each of the enhancer probes since most enhancer-promoter interactions occur within a topologically associating domain (TAD) that is less than 1Mb in size [39]. From these, we selected the genes that were significantly upregulated in tumor relative to normal as potential targets of these enhancers. For example, we found that the *SPR* gene was a potential target of the enhancer probe cg0416113 (Fig 7). *SPR* (sepiapterin reductase) is located ~177kb upstream of the enhancer probe. A recent study showed that SPR depletion inhibited liver cancer cell proliferation and promoted cancer cell apoptosis *in vivo* [40], suggesting its role as an oncogene. Gene ontology (GO) analyses revealed that target genes potentially regulated by CENPA, FOXM1, and MYBL2 are involved in cell cycle, cellular response to DNA damage stimulus, chromosome organization, and DNA repair (S8A and S8B Table).

**Fig 8:**
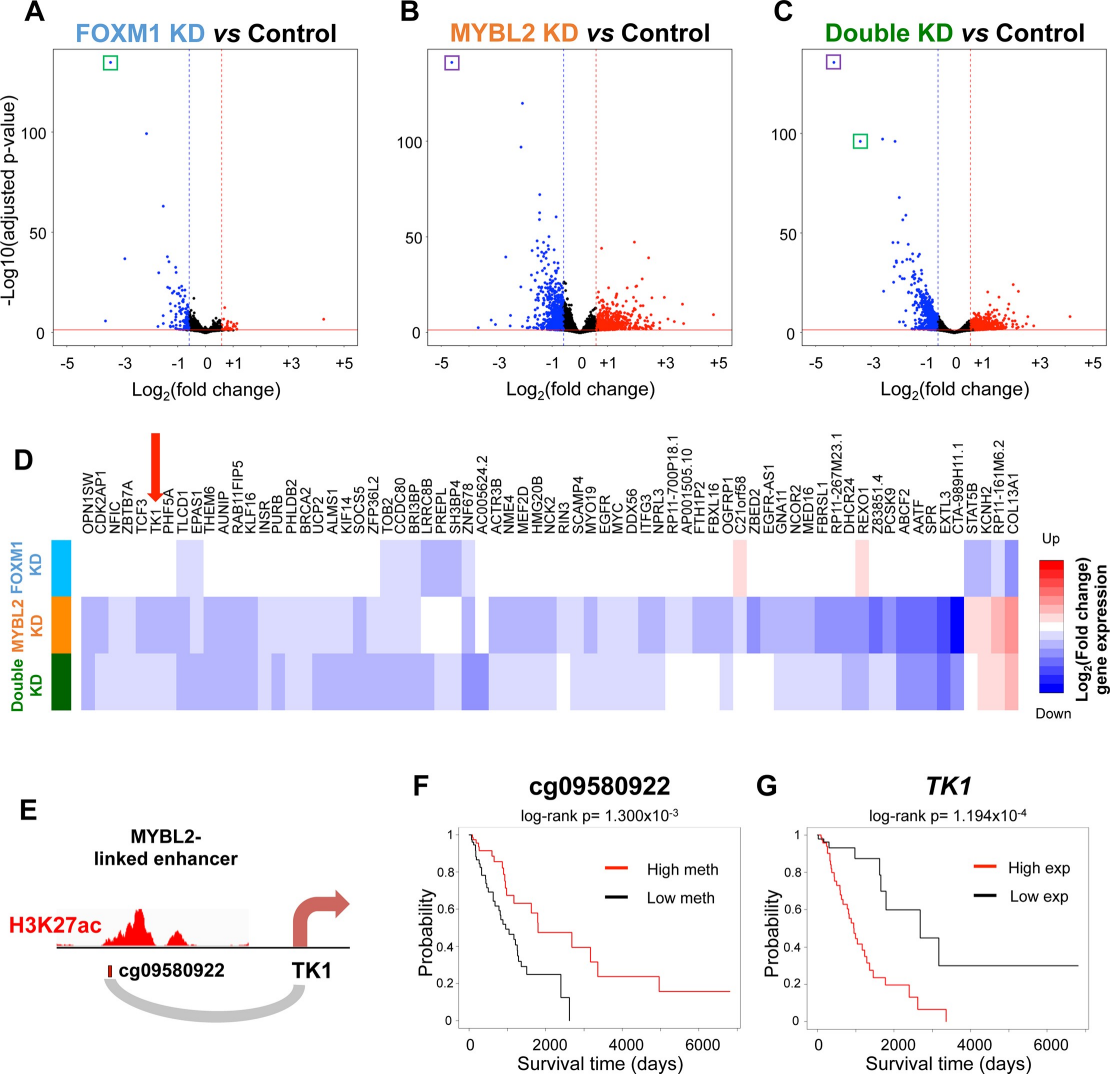
Identification of genes regulated by FOXM1 and MYLB2. Volcano plots showing gene expression changes after knocking down (A) FOXM1 or (B) MYBL2 or (C) Double (both FOXM1 and MYBL2). The knocked down genes (FOXM1 or MYBL2) are highlighted by a green or purple box, respectively. (D) Heatmap displaying fold change expression of significantly downregulated genes in the vicinity of cancer-specific enhancers associated with poor patient survival after FOXM1 (light blue) or MYBL2 (orange) or double (green) knockdown; log2(fold change) were plotted from dark blue to dark red (see S9 Table). Genes shown represent potential target genes within 1 Mb of CENPA/FOXM1/MYBL2-linked enhancers whose activation is significantly associated with poor patient survival. Expression of the gene *TK1* is highlighted by the red arrow. (E) Diagram of A549 H3K27ac mark overlapping the MYBL2-linked probe cg09580922 and its potential target gene *TK1* (see S10 Fig). (F) Kaplan-Meier survival plot comparing differences in survival between LUAD tumor samples with the highest and lowest quartiles of cg09580922 methylation. (G) Kaplan-Meier survival plot comparing differences in survival between LUAD tumor samples with the highest and lowest quartiles of *TK1* expression.

To identify genes and signaling pathways regulated by FOXM1 and MYBL2, known transcription factors, we performed knockdown experiments for FOXM1 and MYBL2 in A549 cells, a LUAD cell line. More than a thousand genes were differentially expressed upon knockdown of either FOXM1 or MYBL2 or both (Fig 8A–8C, S9A–S9C Table). GO analyses of the genes downregulated after knocking down FOXM1 or MYBL2 or both indicated that these genes are involved in cell cycle and cell division, supporting the gene predictions made from degerulated genes near the activated enhancers (S8C–S8E Table). We determined which of the significantly downregulated genes from the siRNA knockdowns were located within 1 Mb of the enhancer probes we had previously linked to these transcriptional regulators (Fig 8D, S9D Table). These genes likely represent the direct target genes of the enhancers.

Of particular interest is the gene *TK1*, which showed a ~40% reduction in expression after MYBL2 knock down (adjusted p = 2.506x10^-7^) (S9B and S9C Table). *TK1*, encoding a protein that plays an important role in thymidine metabolism, is located ~188 kb from the MYBL2-linked enhancer probe cg09580922 on chr17q25.3 (Fig 8E). Low methylation of cg09580922 is strongly associated with poor patient survival (Fig 8F), as is high expression of *TK1* (Fig 8G). The promoter of *TK1* and cg09580922 are both located in the same TAD according to Hi-C maps from both A549 as well as GM12878, another cell line for which a high resolution Hi-C dataset is available (S10 Fig). This suggests that a cancer-specific enhancer potentially regulated by MYBL2 may increase the expression of *TK1*. A complete list of enhancers and their potential target genes confirmed by knockdown experiments and located in the same TAD can be found in S9D Table.

## Discussion

We have developed TENET 2.0, a method to characterize enhancer networks controlled by transcriptional regulators that are potential tumor suppressors or oncogenic drivers. Using H3K27ac ChIP-seq and open chromatin datasets, we identified enhancers active in lung cells. Then, using DNA methylation levels at the identified enhancers in hundreds of normal *vs*. LUAD tissue samples [7], we identified over 32,000 differentially activated enhancers. By integrating DNA methylation and gene expression data, we identified key transcriptional regulators (e.g. NKX2-1, CENPA, FOXM1, and MYBL2) that are linked to many cell-type specific enhancers. We further found that high expression of *CENPA*, *FOXM1*, and *MYBL2* is associated with poor survival in patients with LUAD and with broad enhancer activation in a distinct group of LUAD tumors. We found a subgroup of BRCA tumor samples which also showed activation of BRCA enhancers linked to these three transcriptional regulators, and basal-subtype tumors were particularly enriched in that subgroup. We then identified LUAD-specific enhancers that are linked to the three transcriptional regulators and whose increased activities are correlated with poor survival. For example, the enhancer marked by probe cg09580922 appears to regulate the *TK1* gene, whose high expression is associated with poor patient survival.

TENET 2.0, which now has updated databases, including new algorithms to identify epigenetic traits associated with mortality with greatly decreased computational time, allowed us to identify dysregulated transcriptional regulators and enhancers in LUAD. Key inactivated transcriptional regulators, which are potential tumor suppressors, include NKX2-1 (Fig 3). Low expression of NKX2-1 was observed in a subgroup of LUAD samples (S8A Fig) and was linked to over a hundred inactivated enhancers. NKX2-1, also known as thyroid transcription factor 1 (TTF1), regulates transcription of genes specific for the thyroid and lung. NKX2-1 is reported to be involved in lung development, and it inhibits epithelial to mesenchymal transition, supporting its role as a tumor suppressor [41,42]. Besides NKX2-1, we identified that HNF1B, a previously reported tumor suppressor found in other cancer types [33–35], STAT6, and SP100 were inactivated and linked to many silenced enhancers in a subgroup of LUAD (Fig 3, S5A Table).

Of the transcriptional regulators activated in LUAD, CENPA, FOXM1, and MYBL2 were linked to the activation of hundreds of cancer-specific enhancers. These transcriptional regulators are therefore potential cancer driver oncogenes. CENPA, which has a histone-binding domain, directs the assembly of active kinetochores together with centromere-specific-DNA-binding factors. A recent study in cervical and colorectal cancer cells reported that CEPNA can also bind to DNaseI hypersensitive sites [43]. MYBL2 (a.k.a. B-MYB), a member of the MYB family, regulates cell cycle genes by binding to regulatory elements [44]. FOXM1, a member of the Forkhead family of pioneer transcription factors [45], is involved in the proper development of several different organ systems, including the lungs [37]. It has been demonstrated to bind to enhancers in breast cancer cells [46]. Here we showed that CENPA, FOXM1, and MYBL2 are upregulated together, potentially leading to the activation of many cancer-specific enhancers in a subgroup of LUAD. The subgroup of LUAD with both high expression of *CENPA*, *FOXM1*, and *MYBL2* and broad enhancer activation had worse patient survival outcomes. This subgroup also appears to have a higher proportion of smokers, which may be related to the observed epigenomic changes, and higher tumor mutational burden [47]. It has been previously suggested that FOXM1 may act as a regulator for genes involved in DNA damage response and repair [48]. Besides these 3 transcriptional regulators, we identified other key transcriptional regulators, such as TCF24, SOX2, and ZNF695, each linked to over 500 enhancers activated in LUAD (Fig 3), providing many further avenues of investigation.

When we compared our LUAD data with that of a similar analysis of BRCA, *CENPA*, *FOXM1*, and *MYBL2* were also found to be activated, particularly in basal-subtype tumors, supporting the idea that these factors work together in certain cancer subtypes. Previously, we showed that estrogen receptor and FOXA1, which are known to be activated in estrogen receptor-positive breast cancer subtypes (e.g. luminal A, luminal B), are not expressed in the basal subtype, but FOX and MYB motifs are enriched at enhancers in basal-like breast cancer cells [49]. FOXM1 and MYBL2 motifs were enriched at CENPA, FOXM1, and MYBL2-linked enhancers we found in lung cancer cells (91.8% for a FOXM1 motif, 60.3% for an MYBL2 motif) (S10 Table). Interestingly, CENPA, FOXM1, and MYBL2 appear to target different enhancers in BRCA and LUAD, potentially working with different co-factors [50]. In spite of this difference, GO analysis of potential target genes for these enhancers revealed that both sets regulate similar cellular processes, including cell cycle control and DNA repair (S8A and S8B Table). Further studies to elucidate the function of these transcriptional regulators in tumor subgroups are needed to better understand their role in epigenetic deregulation of cancer cells.

Previous studies had implicated FOXM1 and MYBL2 in lung cancer [51–53], but our analysis documents their profound effects on gene deregulation, potentially affecting hundreds of enhancers. As acquisition of cancer-specific enhancers can drive tumorigenesis [54], identifying key activated enhancers in cancer is highly relevant. Here, we identified 101 LUAD-specific enhancers linked to CENPA, FOXM1, and MYBL2 that show correlations with worse survival (Fig 7, S3B Table). For example, we found that the enhancer probe cg04161113, whose activation (low DNA methylation) is associated with poor survival, is potentially regulating the *SPR* gene, which was recently reported as an oncogene in liver cancer [40].

Using knockdown experiments, we further identified potential target genes of these enhancers, which included genes involved in cell division and cell cycle control. These potential target genes included not only known oncogenes such as *MYC*, *FBXL16*, *PHF5A*, and *KIF14* [55–58] but also genes (e.g. *BRI3BP*, *RAB11FIP5*) which are not yet reported to be involved in lung carcinogenesis (S9 Table). Of the downregulated genes after siRNA treatment, *TK1* was the most significantly associated with survival rates (log-rank p = 1.194x10^-4^). High expression of *TK1* and low methylation of the nearby MYBL2-linked enhancer probe cg09580922 were associated with poor patient survival (Fig 8F and 8G), and both appear to be located in the same TAD (S10 Fig). *TK1* has been investigated as a diagnostic and prognostic biomarker for several types of cancer, including LUAD [59]. Loss of TK1 has been shown to inhibit the growth and metastatic capabilities of LUAD *in vitro* as well as in mice through a reduction in expression of *GDF15* [59].

We have used TENET 2.0 to integrate epigenomic and transcriptomic profiles from hundreds of samples and have identified key transcriptional regulators and enhancers altered in LUAD. The lists of these enhancers, transcriptional regulators, and their potential target genes will be a useful resource for researchers aiming to better understand the molecular mechanisms driving carcinogenesis in different LUAD subgroups. Moreover, our findings may lead to new biomarkers as well as therapies that might target distinct LUAD subgroups associated with poor survival; small molecule inhibitors for MYB family members [60] as well as FOXM1 have been developed but have not yet been tested in lung cancer [61,62]. Importantly, TENET 2.0 can be used to investigate molecular mechanisms underlying any cancer type for which gene expression and epigenetic data are available (http://github.com/suhnrhie/TENET_2.0).

## Materials and methods

### Ethics statement

Remnant human transplant lungs were obtained in compliance with USC Institutional Review Board protocol, approved for the use of human source material in research (HS-07-00660). As donors were deceased and de-identified, no patient consent was obtained or necessary.

### Cell culture

Human lung adenocarcinoma A549 cells (Cat # CRL-185, ATCC, Gaithersburg, MD) were grown at 37°C with 5% CO2 in RPMI 1640 (Cat #10-040-CV, Corning, NY, USA) supplemented with 10% fetal bovine serum (FBS) (Cat # FBS-500, X&Y Cell Culture, MI, USA) and 100 units/ml of penicillin/streptomycin (formulated by Norris Comprehensive Cancer Center Media Core, CA, USA). Human AT2 cells were isolated from remnant transplant lung from deceased de-identified non-smoking donors in compliance with USC Institutional Review Board protocol for the use of human source material in research (HS-07-00660). As donors were deceased and de-identified, no patient consent was obtained or necessary. Lungs were processed as previously described [23]. The three donors were 25, 62, and 67-year-old males who died of non-lung related causes. AT2 cells were isolated from the samples, plated in 50% DMEM/F12 (Cat #D64421, Sigma, MO, USA), 50% DMEM high glucose (Cat #21063, GIBCO, MA, USA), supplemented with 10% FBS, penicillin/streptomycin, 50 ug/ml gentamycin (Cat #G1272, Sigma, MO, USA) and 2.5ug/ml amphotericin (Cat #A2411, Sigma, MO, USA), to allow differentiation to AT1-like cells, and isolated at three different time points (D0, D4, D6) as previously noted [22,23] (S1 Table).

### siRNA knockdown and RNA-seq

A549 cells were transfected in quadruplicate with 100nM of ON-TARGETplus siRNA oglionucleotides for human *FOXM1* (Cat # L-009762-00-005, Dharmacon—Horizon Discovery, UK), *MYBL2* (Cat # L-010444-00-005, Dharmacon—Horizon Discovery, UK), both, or non-targeting control (Cat # D-001810-10-05, Dharmacon—Horizon Discovery, UK), mixed with 5X siRNA buffer (Cat # B-002000-UB-100, Dharmacon—Horizon Discovery, UK) and transfected using DharmaFECT 1 Transfection reagent (Cat # T-2001-01, Dharmacon—Horizon Discovery, UK). Cells were transfected, cultured for 24 hours, and transfected again with the same concentration of siRNA, then incubated for an additional 24 hours before RNA was extracted using the Aurum Total RNA Mini Kit (Cat # 7326820, Bio-Rad, CA, USA). cDNA was synthesized using an iScript cDNA Synthesis Kit (Cat # 1708891, Bio-Rad, CA, USA) and expression levels of *FOXM1* and *MYBL2* were checked with qRT-PCR using SYBR Green Supermix (Cat # 1708886, Bio-Rad, CA, USA) with the listed primers (S11 Table). RNA-seq was performed using 150 bp paired-end sequencing using an Illumina HiSeq 4000 (GENEWIZ, South Plainfield, NJ, USA) for the single gene knockdown experiments, and using 100 bp paired-end sequencing using an Illumina NovaSeq 6000 (MedGenome, Foster City, CA, USA) for the double knockdown. RNA-seq reads were aligned to the human reference genome hg38 using the Genomic Data Commons Bioinformatics mRNA analysis pipeline. Read counts were generated for GENCODE v22 genes [19] using the htseq-count function [63]. Differentially expressed genes were called using DESeq2 [64] with the lfcShrink function [65]. Gene ontology analyses were performed using PANTHER [66] (S8C–S8E Table) (see S1 Methods for more details).

### ChIP-seq

ChIP-seq was performed on the D0, D4, and D6 AECs isolated from the 25-year-old and 62-year-old male subjects using H3K27ac antibody (Cat # 39133, Active Motif, CA, USA), as previously described [22,23]. The ChIP-seq library from the 25-year-old individual was sequenced using 50 bp single-end reads on an Illumina HiSeq 2000 (S1 Table). Two technical replicates of A549 H3K27ac ChIP-seq data and two replicates of H3K27ac ChIP-seq data from lung tissue from a 53-year-old female donor generated by the ENCODE Consortium [25,26] were used. H3K27ac ChIP-seq data from two additional lung tissue samples from 30-year-old female and 3-year-old male donors generated by the ROADMAP Consortium [67,68] were also included (S1 Table). Finally, H3K27ac ChIP-seq data collected from 12 lung cancer lines from the DBTSS were downloaded and processed as well [27]. ChIP-seq reads were aligned to the human reference genome hg38 and reproducible peaks were called, following the ENCODE ChIP-seq pipeline [69] (see S1 Methods).

### ATAC-seq

Intact nuclei from D0 AT2 cells were isolated from the 67-year-old male subject utilizing the protocol from Buenrostro et al. [70]. Briefly, intact nuclei were isolated and incubated with Tn5 transposase (Cat # FC-121-1030, Illumina, CA, USA). The transposed DNA was extracted and was amplified with PCR using NEBNext High-Fidelity PCR Master Mix (Cat # M0541S, New England Biolabs, MA, USA) and the resulting library was purified using a bead clean with AMPure XP Magnetic Beads (Cat # A63880, Beckman Coulter, CA, USA) and quality control was performed using a BioAnalyzer High-Sensitivity DNA Analysis kit (Cat # 5067–4626, Agilent, CA, USA). Data was sequenced as 75 bp paired-end reads on an Illumina HiSeq 2000. ATAC-seq data were processed using the ENCODE ATAC-seq pipeline (https://www.encodeproject.org/atac-seq/) (see S1 Methods). In addition, ATAC-seq peaks from 34 LUAD tissue samples were downloaded and lifted over to the hg38 reference genome using the LiftOver tool available in the UCSC genome browser (https://genome.ucsc.edu/cgi-bin/hgLiftOver) for TENET 2.0 analyses [30]. ATAC-seq peaks from an additional 22 LUAD tissue samples were added [29] along with peaks from the PC-9 LUAD cell line [28] (S1 Table).

### DNase-seq

Peaks of DNaseI hypersensitive sites in PC-9 and A549 cells processed by the ENCODE consortium were acquired [25,26]. Those from A549 cells aligned to the hg19 human reference genome were lifted over to the hg38 reference genome using the LiftOver tool available in the UCSC genome browser (https://genome.ucsc.edu/cgi-bin/hgLiftOver) (S1 Table).

### TENET program update and settings

Here we improved the original TENET program [14] and developed TENET 2.0. TENET 2.0 uses a new reference genome (hg38) and gene annotation dataset (GENCODE v22) which covers >60,000 transcripts [19]. It also includes a new dataset of 1,639 validated human transcription factors [20], the processing speed is increased, and useful functions were added to identify enhancers, genes, and tumor subgroups associated with survival. For enhancer analysis, we utilized H3K27ac ChIP-seq, ATAC-seq and DNase I hypersensivite site datasets. RNA-seq data along with DNA methylation data were downloaded for BRCA and LUAD samples from the TCGA [7,71] using the TCGAbiolinks package [72] (see S1 Methods for more details on enhancer analysis, TCGA datasets, and TENET 2.0 program). TENET 2.0 program is available at http://github.com/suhnrhie/TENET_2.0.

### Heatmaps

For Fig 4A, unsupervised clustering was performed and for Fig 2D, pairwise correlation coefficients were calculated between each of the top LUAD transcriptional regulators identified and an unsupervised clustering was performed. For Fig 6 and S8 Fig, heatmaps were generated and unsupervised clustering was performed. DNA methylation levels (β) ranging from 0 (unmeth) to 1 (meth) were plotted. Continuous variables, including gene expression, patient age, and tumor purity, were scaled using the function (X—X_min_)/(X_max_—X_min_) with values equal to zero set to the minimum, non-zero value. Tumor purity values, including leukocytes unmethylation for purity, and overall derived consensus purity, were obtained from the Tumor purity dataset available from TCGAbiolinks package [72] (see S1 Methods).

### Expression/correlation analyses

Expression values of key oncogenic transcriptional regulators from the adjacent normal and LUAD tumor samples were plotted, and Student’s t-tests were performed to compare differential expression between normal and tumor groups. An one-way ANOVA analysis was performed to assess overall differences in transcriptional regulator expression between the smoking groups (67 never smokers, 278 former smokers, and 106 current smokers) and a Tukey Honest Significant Differences test was performed to assess significant differences between individual groups. Linear regression models were fit to predict expression of *CENPA*, *FOXM1* and *MYBL2* with respect to variables recorded for sample clinical information in the TCGA, including sample type, sex, age, smoking history, total pack years smoked, and race for samples which contained complete information for these variables. Independent RNA-seq data from 728 lung tumor tissues generated as part of the ORIEN were used to validate our correlation analyses. The correlation analyses were performed using the normalized RSEM values calculated following the ORIEN Total Cancer Care protocol (http://www.oriencancer.org/) accessed in May of 2020 [73–75] (S2 Fig).

### Survival analyses

Survival analyses were performed comparing prognosis of patients with the highest and lowest quartiles of *CENPA*, *FOXM1* and *MYBL2* expression, linked-enhancer probe DNA methylation levels. Patient survival from samples within the "highly linked" group to those without any links to *CENPA*, *FOXM1*, and *MYBL2* were also compared. Survival plots from Kaplan-Meier Plotter were performed on their website (https://kmplot.com/analysis/) [36] (see S1 Methods).

### Genetic alteration and mutation count analysis

Genetic alteration data for LUAD samples in the TCGA PanCancer Atlas was downloaded from the cBioPortal [73,76] by selecting a query for mutations and putative copy-number alterations from GISTIC (https://software.broadinstitute.org/cancer/cga/gistic) for KRAS, EGFR, NF1, and BRAF. 445 of the 453 LUAD tumor samples in the TENET dataset contained information for these four alterations. Samples which were listed as having a "putative driver" mutation, amplification, deletion, or a fusion of each of the genes in this dataset were recorded as being positive for an alteration to that gene. Total mutation count data containing information for 447 of the 453 LUAD tumor samples was also downloaded from the cBioPortal repository [73,76].

### Identification of potential target genes for CENPA/FOXM1/MYBL2-linked probes in LUAD and BRCA

Student’s t-tests were performed for all genes in the LUAD and BRCA datasets, comparing expression in the tumor *vs*. normal samples. Genes that were significantly differentially expressed (fdr-corrected p<0.05) and upregulated specifically in the tumor samples were selected for further gene ontology (GO) analyses (S8A and S8B Table) (see S1 Methods).

### Motif analysis

Minmeme motif files for FOXM1 or MYBL2, based on ChIP-seq experiments (3 from GSM12878 cells, MCF-7 cells, and SK-N-SH cells for FOXM1 and 1 from HepG2 cells for MYBL2), were downloaded from Factorbook (http://factorboook.org) in August of 2019, Additional minmeme motif files for FOXM1 and MYBL2 were downloaded from the HOCOMOCO v11 database [77]. Motif files we used are listed in S10 Table. Using these motif files and FIMO program [78], we scanned DNA sequences within 1,117 bp, equivalent to half the average enhancer size as calculated from the lung enhancer regions (S2A Table), of FOXM1, MYBL2, or CENPA-linked enhancers (n = 1,338).

### Hi-C analysis

Using “ENCODE3-iced” data from A549 cells [25] and “Rao_2014-raw” data from GM12878 cells [79], Hi-C heatmaps (25kb resolution, hg38) in S10 Fig were created from the 3D genome browser (http://promoter.bx.psu.edu/hi-c/view.php). Both datasets were processed and normalized using the pipeline, described in Wang et al. [80]. TAD information from A549 and GM12878 cells was downloaded from ENCODE and Rao et al. [79], respectively (S1 Table).

## Supporting information

S1 Fig TENET 2.0 pictoral workflow. (A) DNA methylation levels of enhancer probes are used to assess differential activity of transcriptional regulator-linked enhancers. Enhancer probes are identified using H3K27ac ChIP-seq peaks overlapping with regions of open chromatin. Probes intersecting both of these regions are subsetted to those >1.5kb from GENCODE v22-annotated transcription start sites to avoid promoter regions. (B) TENET classifies enhancer probes based on their differential activity as measured by methylation level in normal *vs*. tumor samples. Methylated and unmethylated probes represent enhancers that are uniformly inactive and active, respectively. Hypermethylated probes represent enhancers that are inactive in cancer samples. These probes possess a low level of mean methylation in normal samples, but higher levels of methylation in a subset of tumor samples. Conversely, hypomethylated probes represent enhancers that are active in cancer samples, showing a decreased level of methylation in tumor *vs*. normal lung samples. (C) Analyses are focused on transcriptional regulators that are overexpressed in LUAD, resulting in increased activity of their regulated enhancer regions as represented by decreased DNA methylation. The expression of each transcriptional regulator and DNA methylation of each enhancer probe are assessed to find "linked" pairs with increased expression of the transcriptional regulator and decreased methylation of the probe in a subset of the tumor samples, relative to normal samples. (D) Transcriptional regulators with the most linked enhancers are of interest for study because they are more likely to have large-scale effects on genome-wide expression patterns. TENET 2.0 also includes new functions to identify tumor subgroups based on differences in the activation of enhancers linked to the top transcriptional regulators using heatmaps (E) and association with patient survival (F) as well as potential target genes of enhancers using topologically associating domain (TAD) information (G).
(TIF)

S2 Fig Correlation analyses of key transcriptional regulators activated in lung cancer using ORIEN datasets. (A) Using ORIEN gene expression datasets from lung tumor tissue samples (n = 728), TR gene expression correlation analyses were performed. Barplots show the top 5 most correlated transcriptional regulators for CENPA (top), FOXM1 (middle), and MYBL2 (bottom). (B) TR gene expression scatterplots are shown for *FOXM1 vs*. *MYBL2* (top), *FOXM1 vs*. *CENPA* (middle), and *CENPA vs*. *MYBL2* (bottom).
(TIF)

S3 Fig Expression of top 12 transcriptional regulators activated in LUAD. Boxplots of expression of remaining 9 of top 12 oncogenic transcriptional regulators in 453 TCGA LUAD tumor and 21 adjacent normal samples (*CENPA*, *FOXM1*, or *MYBL2* are shown in Fig 5A). All genes were upregulated in LUAD tumors, but none as strongly as *CENPA*, *FOXM1*, or *MYBL2*.
(TIF)

S4 Fig Survival analysis of top 12 transcriptional regulators activated in LUAD. Kaplan-Meier survival plots comparing differences in survival between samples with the highest and lowest quartiles of expression of the remaining 9 of top 12 cancer-specific transcriptional regulators by number of linked enhancers (*CENPA*, *FOXM1*, and *MYBL2* are shown in Fig 5B). Survival was compared using TCGA LUAD samples.
(TIF)

S5 Fig Replication of association of expression of highly-linked oncogenic transcriptional regulators with patient survival in LUAD using Kaplan-Meier Plotter. Kaplan-Meier survival plots comparing differences in survival between samples with the highest and lowest quartiles of expression of the top 12 oncogenic transcriptional regulators in LUAD cases using Kaplan-Meier Plotter (https://kmplot.com/analysis/) [36]. Again, expression of *CENPA*, *FOXM1*, and *MYBL2* was the most strongly associated with patient survival amongst these transcriptional regulators.
(TIF)

S6 Fig Smoking history is associated with *CENPA*, *FOXM1*, and *MYBL2* expression in TCGA samples. (A) Boxplots of *CENPA*, *FOXM1*, and *MYBL2* expression in 453 TCGA LUAD tumor samples stratified by smoking history. Tumor samples from current smokers had significantly higher expression of all three transcriptional regulator genes than those from former smokers and individuals who had never smoked (significant Tukey HSD p-values displayed; ***p<0.005). (B) Boxplots show *CENPA*, *FOXM1*, and *MYBL2* expression in all 453 TCGA LUAD tumor samples stratified by median total mutational count. Samples with higher mutational burden had higher expression of these transcriptional regulators.
(TIF)

S7 Fig Association of total active enhancer links with three expression of three activated transcriptional regulators and common LUAD mutations. (A) Boxplots show the total number of links to activated enhancers on a per sample basis in LUAD samples with the highest quartile and lowest quartile of *CENPA*, *FOXM1*, and *MYBL2* and expression. (B) Boxplots display differences in the total number of links to activated enhancers on a per sample basis in LUAD samples stratified by the presence and absence of a *KRAS* (right) or *EGFR* alteration (left), and with and without the highest quartile of expression of *FOXM1* (right) or *MYBL2* (left). There is a significant difference in the number of links between samples in the highest quartile of expression *vs*. the other three quartiles for both transcriptional regulators regardless of their alteration status, but no significant difference in the number of links between samples with and without either alteration but with the same expression level (Significant Tukey HSD p-values displayed; * = p<0.05, ** = p<0.01, *** = p<0.005).
(TIF)

S8 Fig Association of links to CENPA, FOXM1, MYBL2-linked enhancers with clinical data and subgroup analysis. (A) Heatmap of DNA methylation β-values for *CENPA*/*FOXM1/MYBL2*-linked lung cancer-specific enhancers (n = 1,338) for normal and LUAD tissue samples. From top to bottom, samples are plotted with the age, sex, and cancer stage of the patients, smoking history status, expression of additional identified TRs *TCF24*, *SOX2*, and *NKX2-1*, leukocyte and overall tumor purity, presence of *KRAS*, *EGFR*, *NF1* and *BRAF* alterations, log2-transformed mutational count, and sample link status. (B) Chi-square test results comparing smoking history in the more active cluster **b**, to the less active cluster **a** from Fig 6A. There is a much greater proportion of current smokers in cluster b than in cluster a. (C) t-test results comparing mean total mutation count of samples in cluster b to cluster a. Samples in cluster b have a significantly higher mean tumor burden than samples in cluster a. (D) Univariate Kaplan-Meier survival plot comparing difference in survival between the very highly-linked samples (marked in red in the link status bar), and samples that do not possess any links to CENPA/FOXM1/MYBL2-linked probes (marked in blue in the link status bar).
(TIF)

S9 Fig Key transcriptional regulators identified in LUAD *vs*. BRCA and comparison of CENPA/FOXM1/MYBL2-linked probes in each dataset. (A) Barplot of the top 12 transcriptional regulators by number of links to activated enhancers identified using TENET 2.0 in LUAD (left) and BRCA (right). (B) Venn diagrams display overlap of probes linked to CENPA, FOXM1, and MYBL2 and (C) all hypomethylated probes in the LUAD *vs*. BRCA analyses. There is a considerably higher percentage of overlap between all hypomethylated probes than for probes linked only to CENPA, FOXM1, or MYBL2.
(TIF)

S10 Fig Hi-C diagrams from A549 and GM12878 cells showing the cg09580922 and *TK1* genomic region. Hi-C diagrams of A549 cells (top) and GM12878 cells (bottom) show the genomic context of the *TK1*/cg09580922 locus (middle) from chr17:77125000–79625000. In both cell lines, TAD boundaries (lower middle) show that both *TK1* and cg09580922 are located in the same TAD.
(TIF)

S1 Table Datasets used in this study. (XLSX)

S2 Table List of enhancer and open chromatin regions identified in lung for this study. A) List of enhancer regions. B) List of open chromatin regions.
(XLSX)

S3 Table List of HM450 enhancer probes. A) List of HM450 probes by enhancer type in LUAD. B) List of CENPA/FOXM1/MYBL2-linked enhancer probes in LUAD. C) List of HM450 probes by enhancer type in BRCA. D) List of hypomethylated enhancer probes in BRCA.
(XLSX)

S4 Table List of TCGA IDs of LUAD and BRCA samples included in this study. (XLSX)

S5 Table Lists of top transcriptional regulators identified by TENET 2.0. A) List of top inactivated transcriptional regulator genes in LUAD. B) List of top inactivated transcriptional regulator genes in BRCA. C) List of top activated transcriptional regulator genes in LUAD. D) List of top activated transcriptional regulator genes in BRCA. E) Order of transcriptional regulator genes and enhancer probes as rows/columns for Fig 4A. F) Order of transcriptional regulator genes for Fig 4B.
(XLSX)

S6 Table Regression analysis of *CENPA/FOXM1/MYBL2* expression in LUAD. (XLSX)

S7 Table Lists of CENPA/FOXM1/MYBL2-linked enhancer probes in LUAD or BRCA. (XLSX)

S8 Table Gene Ontology categories enriched in potential target genes of CENPA, FOXM1, and MYBL2. A) GO analysis of genes linked to CENPA/FOXM1/MYBL2 linked enhancer probes in LUAD. B) GO analysis of genes linked to CENPA/FOXM1/MYBL2 linked enhancer probes in BRCA. C) GO analysis of genes significantly downregulated after si*FOXM1* treatment in A549 LUAD cells. D) GO analysis of genes significantly downregulated after si*MYBL2* treatment in A549 LUAD cells. E) GO analysis of genes significantly downregulated after double si*FOXM1* and si*MYBL2* treatment in A549 LUAD cells.
(XLSX)

S9 Table List of differentially expressed genes identified using siRNA knockdown experiments. A) List of differentially expressed genes by si*FOXM1* in A549 LUAD cells. B) List of differentially expressed genes by si*MYBL2* in A549 LUAD cells. C) List of differentially expressed genes by double si*FOXM1* and si*MYBL2* in A549 LUAD cells. D) Potential genes regulated by CENPA/FOXM1/MYBL2-linked enhancers confirmed by siRNA experiment in A549 LUAD cell line.
(XLSX)

S10 Table List of FOXM1 and MYBL2 motifs. (XLSX)

S11 Table List of primer sequences used for RTqPCR. (XLSX)

S1 Methods (DOCX)
