# Supplementary material for: TENET 2.0: Identification of key transcriptional regulators and enhancers in lung adenocarcinoma
Source: PLoS Genet. 2020 Sep 14;16(9):e1009023. doi: 10.1371/journal.pgen.1009023 (PMC7515200; doi:10.1371/journal.pgen.1009023)
Supplement: S1 Methods — (DOCX) [file pgen.1009023.s022.docx]

**S1_Methods**

**SiRNA knockdown and RNA-seq:** A549 cells were transfected in quadruplicate with 100nM of ON-TARGETplus siRNA oglionucleotides for human FOXM1 (Cat # L-009762-00-005, Dharmacon - Horizon Discovery, UK), MYBL2 (Cat # L-010444-00-005, Dharmacon - Horizon Discovery, UK), both, or non-targeting control (Cat # D-001810-10-05, Dharmacon - Horizon Discovery, UK), mixed with 5X siRNA buffer (Cat # B-002000-UB-100, Dharmacon - Horizon Discovery, UK) and transfected using DharmaFECT 1 Transfection reagent (Cat # T-2001-01, Dharmacon - Horizon Discovery, UK). Cells were transfected, cultured for 24 hours, and transfected again with the same concentration of siRNA, then incubated for an additional 24 hours before RNA was extracted using the Aurum Total RNA Mini Kit (Cat # 7326820, Bio-Rad, CA, USA). cDNA was synthesized using an iScript cDNA Synthesis Kit (Cat # 1708891, Bio-Rad, CA, USA) and expression levels of *FOXM1* and *MYBL2* were checked with qRT-PCR using SYBR Green Supermix (Cat # 1708886, Bio-Rad, CA, USA) with the listed primers (S11 Table). RNA-seq was performed using either 150 bp or 100 bp paired-end sequencing on Illumina HiSeq 4000 or NovaSeq 6000. RNA-seq reads were aligned to the human reference genome hg38 using the Genomic Data Commons Bioinformatics mRNA analysis pipeline (https://docs.gdc.cancer.gov/Data/Bioinformatics_Pipelines/Expression_mRNA_Pipeline/). In brief, the two-pass alignment method was performed using STAR v2.6.0a [1] using settings as specified by the pipeline. Read counts were generated for GENCODE v22 genes [2] using the htseq-count function from HTSeq v0.11.1 [3]. Differentially expressed genes between control and each set of siRNA knockdown samples were called using DESeq2 [4] along with the lfcShrink function from the apeglm package [5], using 29,515 expressed genes for single knockdown analysis, or 28,035 genes for the double knockdown analysis, after removing genes with total exon lengths less than 150 bp or that were unexpressed in all four replicates of either the control, siFOXM1, or siMYBL2 treated cells.

**ChIP-seq:** ChIP-seq was performed on the D0, D4, and D6 alveolar epithelial cells (AECs) isolated from the 25-year-old and 62-year-old male individuals as previously described [6,7]. The ChIP-seq library from the 25-year-old individual was created after performing QC and sequenced using 50bp single-end reads on an Illumina HiSeq 2000 at the University of Southern California Epigenome Center, as previously done [6,7] (S1 Table). Fastq files for two technical replicates of A549 H3K27ac ChIP-seq data and two replicates of H3K27ac ChIP-seq data from lung tissue from a 53-year-old female donor generated by the ENCODE Consortium [8,9] were downloaded from Gene Expression Omnibus (GEO). The two replicates of this ENCODE lung tissue data were pooled together in order to analyze them in the same manner as the other lung samples described subsequently. Fastq files for H3K27ac ChIP-seq data from two additional lung tissue samples from 30-year-old female and 3-year-old male donors generated by the ROADMAP Consortium [10,11] were also acquired from GEO. Finally, H3K27ac ChIP-seq datasets collected from 12 different LUAD cell lines were acquired from the DataBase of Transcriptional Start Sites (DBTSS) [12] (S1 Table). All of ChIP-seq datasets were processed using the ChIP-seq pipeline used by the ENCODE consortium [13]. In brief, bwa v.0.7.17 [14] was used to align the reads to the human reference genome hg38, trimming the first 5 base pairs of each read, allowing for two mismatches. Duplicate reads were marked using Picard MarkDuplicates v.2.12.0 (https://broadinstitute.github.io/picard/), and the duplicated reads and reads with mapping quality lower than 30 were removed using samtools v.1.9 [15]. Cross correlation and tagalign files were created using run_spp_nodups [13,16]. Peaks and signal tracks were generated using the Macs2 v.2.1.2 peak caller [17]. For peak calling, the callpeak function was used with following settings (a p-value cutoff set to 0.01, no shift selected, an extension size calculated from cross correlation), and a pileup signal file was created. This pileup signal file was used by the bdgcmp function to generate the bigwig signal files for ChIP reads. For the ChIP-seq data from ENCODE A549 cells, to ensure the called peaks were reproducible, irreproducible discovery rate (idr) analysis was performed using idr v.2.0.2 with a threshold set to 5% (https://github.com/nboley/idr).

**Creation of enhancer dataset:** Called H3K27ac ChIP-seq peaks were combined and pooled within their respective cell type or data source. To ensure inclusion of only reproducible peaks for the final enhancer dataset, peaks from the datasets within each group were overlapped. Only peaks found in at least two of the datasets in a given grouping were included in the final datasets. The final overlapping peaks from each group were pooled with each other and were collapsed to form a single file using the reduce function from the GenomicRanges package in R 3.6.0 [18]. A total of six H3K27ac datasets were individually grouped: 1) peaks from D0 AECs, 2) peaks from D4 AECs, 3) peaks from D6 AECs, 4) peaks from three normal lung tissue samples obtained from from ENCODE and ROADMAP, 5) idr-passed peaks from A549 LUAD cell line from ENCODE, and 6) peaks from 12 LUAD cell lines obtained from DBTSS. Open chromatin datasets were also individually grouped: 1) ATAC-seq peaks from D0 AECs (top 25,000 peaks ranked by p-value), 2) ATAC-seq peaks from 34 LUAD tissue samples from Wang *et al.* (peaks were lifted over to the hg38 reference genome using the LiftOver tool available in the UCSC genome browser (<https://genome.ucsc.edu/cgi-bin/hgLiftOver>) [19], 3) ATAC-seq peaks from 22 LUAD tissue samples collected by The Cancer Genome Atlas [20], 4) ATAC-seq peaks from PC-9 LUAD cell line [21], 5) DNaseI hypersensitive sites from PC-9 LUAD cell line and 6) DNaseI hypersensitive sites from A549 LUAD cell line. DNase-seq peaks were downloaded from ENCODE [8,9] and lifted over to the hg38 reference genome. All of datasets we used to call enhancer and open chromatin regions are listed in S1 Table. Comprehensive genomic coordinates of enhancer and open chromatin regions (hg38) found in lung and LUAD are listed in S2A-B Table.

**TCGA datasets:** Gene expression RNA-seq data in the form of log2-transformed fragments per kilobase of transcript per million mapped reads, upper quartile normalized (FPKM-UQ), along with DNA methylation data in the form of β-values for HM450 probes were downloaded for BRCA and LUAD samples from the TCGA database [22,23] using the TCGAbiolinks package [24]. Samples with matching gene expression and DNA methylation data were identified. For patients with multiple tumor samples, one pair of matched methylation and expression data was selected at random and the additional samples from those patients were removed. This final dataset consisted of gene expression data for 60,483 GENCODE v22-annotated genes and DNA methylation data for 396,095 HM450 probes, from a total of 453 tumor and 21 normal LUAD samples along with 776 tumor and 83 normal BRCA samples (S4 Table). These datasets along with above enhancer and open chromatin datasets were utilized for TENET 2.0.

**TENET 2.0 program update and settings:** The basic methodology of the TENET was not changed in TENET 2.0 [25]. However, TENET 2.0 uses a new reference genome (hg38) and gene annotation dataset (GENCODE v22) which covers >60,000 transcripts [2]. It also includes a new dataset of 1,639 human transcription factors as transcriptional regulators. We note that these regulators may affect gene expression through different mechanisms; a given protein may have transcription factor-like activity by binding DNA and preventing a canonical transcription factor from binding, not necessarily directly regulating the transcription of target genes [26]. The processing speed is increased, and useful functions were added to identify enhancers, genes, and tumor subgroups associated with survival (see below). For LUAD enhancer analysis, we opted to utilize the reference H3K27ac ChIP-seq, ATAC-seq and DNaseI hypersensivite site datasets in addition to lung datasets (S1 Table). We used the default parameter settings except that we set the methcutoff at 0.7, and the hypomethcutoff at 0.6. Conversely, the unmethcutoff was set to 0.3, and the hypermethcutoff at 0.4, and the minTumor parameter was set at the default of 5. For these analyses, we elected not to use the purity parameters, but investigated the purity effects by visualizing in heatmaps (S8A Fig). For BRCA enhancer analysis, we used the previously used default parameter settings [25].

**Heatmaps:** For Fig 4A, an unsupervised clustering was performed using the binary method for the distance matrix computation, and Ward's actual method for hierarchical clustering. For Fig 4B, pairwise correlation coefficients were calculated between each of the top 84 LUAD transcriptional regulators identified and an unsupervised clustering was performed using the Euclidean method for the distance matrix computation, and Ward's actual method for hierarchical clustering. For Fig 6 and S8 Fig, unsupervised clustering was performed in each plot by calculating Euclidean distance and utilization of Ward's actual method for hierarchical clustering. DNA methylation levels (β-value, ranging from 0 (unmeth) to 1 (meth)) were colored using the jet.colors function. Continuous variables, including gene expression, patient age, and tumor purity, were scaled using the function (X - X_min_)/(X_max_ - X_min_), with values equal to zero set to the minimum, non-zero value. Tumor purity values, including leukocytes unmethylation for purity, and overall derived consensus purity, were obtained from the Tumor.purity dataset available from TCGAbiolinks package [24]. Other values were obtained from the TCGA clinical data for the samples. For highly linked samples, we first defined links between genes and enhancer probe methylation using a population-based method. To assess if an individual sample is likely to harbor a specific hypomethylated link, for each link of interest, we assessed if the β-value of the probe in individual samples falls below the defined hypomethcutoff (0.6), and if the individual sample possessed significantly higher expression of the linked gene than normal samples by performing a t-test comparing the expression in normal samples to the expression of the gene in the individual tumor sample as a set value (bonferroni-adjusted p<0.05). Links were calculated on a per sample basis across all identified hypomethylated probes to transcriptional regulator links, and across just the probes linked to CENPA, FOXM1, and MYBL2; "highly linked" samples were defined as those with more than 290 links (highest 10%). For Fig 8D unsupervised clustering was performed on the 69 genes of interest (those significantly downregulated in at least one siRNA knockdown experiment and located within 1 Mb of a CENPA, FOXM1, or MYBL2-linked probe with reduced methylation associated with poor LUAD patient survival) by calculating Euclidean distance and utilization of complete method for hierarchical clustering. Log2-transformed fold change values were plotted for each gene as calculated by DESeq2 compared to each experiment’s respective control samples.

**Survival analyses:** Survival analyses were performed comparing prognosis of patients with the highest and lowest quartiles of *CENPA*, *FOXM1*, and *MYBL2* expression, samples with the highest and lowest quartiles of linked-enhancer probe DNA methylation levels, as well as samples within the "highly linked" group to those without links to *CENPA*, *FOXM1*, and *MYBL2* as described above, using a version of the TCGAanalyze_survivalKM function from the TCGAbiolinks package [24] in R 3.6.0. The function makes use of TCGA patient survival information to perform univariate Kapan-Meier survival analyses and assess significant differences with a log-rank test. Although the base function performs analysis with gene expression data, we have modified it to provide the same analysis using patient methylation data, or the sample identifiers in our group of interest. We also made slight modifications to the aesthetics of the output graphs. Survival plots from Kaplan-Meier Plotter were performed on their website (<https://kmplot.com/analysis/>) [27]. We assessed survival by restricting the analysis to lung adenocarcinoma samples then compared samples with the highest and lowest quartile of expression of array probes for CENPA, MYBL2, and FOXM1. Displayed figures reflect the output graphs for the single probe listed for each transcriptional regulator.

**Gene ontology analyses:** Gene ontology (GO) analyses were performed using PANTHER [28]. "GO biological process complete" was selected as an annotation dataset, and all significant terms after FDR correction were listed for each analysis. To analyze cellular processes potentially regulated by CENPA-, FOXM1-, or MYBL2-linked probes in LUAD *vs.* BRCA we compared all genes within 1 Mb of these probes that were significantly upregulated (fdr p<0.05) in tumor samples *vs.* normal samples using all genes expressed in at least 50% of tumor samples from each dataset as a reference group (S8A-B Table). To analyze the effect of siRNA knockdown of FOXM1 or MYBL2 or both, we compared the significantly downregulated genes after FOXM1 and MYBL2 knockdown, respectively, using the 29,515 expressed genes analyzed in the RNA-seq for FOXM1 and MYBL2 single knockdowns, or 28,035 expressed genes in the double knockdown experiment as reference groups (S8C-E Table).

**SUPPLEMENTARY REFERENCES**

1. Dobin A, Davis CA, Schlesinger F, Drenkow J, Zaleski C, Jha S, et al. STAR: Ultrafast universal RNA-seq aligner. Bioinformatics. 2013;29: 15–21. doi:10.1093/bioinformatics/bts635

2. Wright JC, Mudge J, Weisser H, Barzine MP, Gonzalez JM, Brazma A, et al. Improving GENCODE reference gene annotation using a high-stringency proteogenomics workflow. Nat Commun. 2016;7: 1–11. doi:10.1038/ncomms11778

3. Anders S, Pyl PT, Huber W. HTSeq-A Python framework to work with high-throughput sequencing data. Bioinformatics. 2015;31: 166–169. doi:10.1093/bioinformatics/btu638

4. Love MI, Huber W, Anders S. Moderated estimation of fold change and dispersion for RNA-seq data with DESeq2. Genome Biol. 2014;15: 1–21. doi:10.1186/s13059-014-0550-8

5. Zhu A, Ibrahim JG, Love MI. Heavy-Tailed prior distributions for sequence count data: Removing the noise and preserving large differences. Bioinformatics. 2019;35: 2084–2092. doi:10.1093/bioinformatics/bty895

6. Yang C, Stueve TR, Yan C, Rhie SK, Mullen DJ, Luo J, et al. Positional integration of lung adenocarcinoma susceptibility loci with primary human alveolar epithelial cell epigenomes. Epigenomics. 2018;10: 1167–1187. doi:10.2217/epi-2018-0003

7. Marconett CN, Zhou B, Rieger ME, Selamat SA, Dubourd M, Fang X, et al. Integrated Transcriptomic and Epigenomic Analysis of Primary Human Lung Epithelial Cell Differentiation. PLoS Genet. 2013;9: 1–14. doi:10.1371/journal.pgen.1003513

8. Dunham I, Kundaje A, Aldred SF, Collins PJ, Davis CA, Doyle F, et al. An integrated encyclopedia of DNA elements in the human genome. Nature. 2012;489: 57–74. doi:10.1038/nature11247

9. Davis CA, Hitz BC, Sloan CA, Chan ET, Davidson JM, Gabdank I, et al. The Encyclopedia of DNA elements (ENCODE): Data portal update. Nucleic Acids Res. 2018;46: D794–D801. doi:10.1093/nar/gkx1081

10. Lister R, Pelizzola M, Dowen RH, Hawkins RD, Hon G, Tonti-Filippini J, et al. Human DNA methylomes at base resolution show widespread epigenomic differences. Nature. 2009;462: 315–322. doi:10.1038/nature08514

11. Bernstein BE, Stamatoyannopoulos JA, Costello JF, Ren B, Milosavljevic A, Meissner A, et al. The NIH roadmap epigenomics mapping consortium. Nat Biotechnol. 2010;28: 1045–1048. doi:10.1038/nbt1010-1045

12. Suzuki A, Kawano S, Mitsuyama T, Suyama M, Kanai Y, Shirahige K, et al. DBTSS/DBKERO for integrated analysis of transcriptional regulation. Nucleic Acids Res. 2018;46: D229–D238. doi:10.1093/nar/gkx1001

13. Landt SG, Marinov GK, Kundaje A, Kheradpour P, Pauli F, Batzoglou S, et al. ChIP-seq guidelines and practices of the ENCODE and modENCODE consortia. Genome Res. 2012;22: 1813–1831. doi:10.1101/gr.136184.111

14. Li H, Durbin R. Fast and accurate short read alignment with Burrows-Wheeler transform. Bioinformatics. 2009;25: 1754–1760. doi:10.1093/bioinformatics/btp324

15. Li H, Handsaker B, Wysoker A, Fennell T, Ruan J, Homer N, et al. The Sequence Alignment/Map format and SAMtools. Bioinformatics. 2009;25: 2078–2079. doi:10.1093/bioinformatics/btp352

16. Kharchenko P V., Tolstorukov MY, Park PJ. Design and analysis of ChIP-seq experiments for DNA-binding proteins. Nat Biotechnol. 2008;26: 1351–1359. doi:10.1038/nbt.1508

17. Zhang Y, Liu T, Meyer CA, Eeckhoute J, Johnson DS, Bernstein BE, et al. Model-based analysis of ChIP-Seq (MACS). Genome Biol. 2008;9. doi:10.1186/gb-2008-9-9-r137

18. Lawrence M, Huber W, Pagès H, Aboyoun P, Carlson M, Gentleman R, et al. Software for Computing and Annotating Genomic Ranges. PLoS Comput Biol. 2013;9: 1–10. doi:10.1371/journal.pcbi.1003118

19. Wang Z, Tu K, Xia L, Luo K, Luo W, Tang J, et al. The open chromatin landscape of non–small cell lung carcinoma. Cancer Res. 2019;79: 4840–4854. doi:10.1158/0008-5472.CAN-18-3663

20. Corces MR, Granja JM, Shams S, Louie BH, Seoane JA, Zhou W, et al. The chromatin accessibility landscape of primary human cancers. Science (80- ). 2018;362: eaav1898. doi:10.1126/science.aav1898

21. Guler GD, Tindell CA, Pitti R, Wilson C, Nichols K, KaiWai Cheung T, et al. Repression of Stress-Induced LINE-1 Expression Protects Cancer Cell Subpopulations from Lethal Drug Exposure. Cancer Cell. 2017;32: 221-237.e13. doi:10.1016/j.ccell.2017.07.002

22. Koboldt DC, Fulton RS, McLellan MD, Schmidt H, Kalicki-Veizer J, McMichael JF, et al. Comprehensive molecular portraits of human breast tumours. Nature. 2012;490: 61–70. doi:10.1038/nature11412

23. Collisson EA, Campbell JD, Brooks AN, Berger AH, Lee W, Chmielecki J, et al. Comprehensive molecular profiling of lung adenocarcinoma: The cancer genome atlas research network. Nature. 2014;511: 543–550. doi:10.1038/nature13385

24. Colaprico A, Silva TC, Olsen C, Garofano L, Cava C, Garolini D, et al. TCGAbiolinks: An R/Bioconductor package for integrative analysis of TCGA data. Nucleic Acids Res. 2016;44: e71. doi:10.1093/nar/gkv1507

25. Rhie SK, Guo Y, Tak YG, Yao L, Shen H, Coetzee GA, et al. Identification of activated enhancers and linked transcription factors in breast, prostate, and kidney tumors by tracing enhancer networks using epigenetic traits. Epigenetics and Chromatin. 2016;9: 1–17. doi:10.1186/s13072-016-0102-4

26. Lambert SA, Jolma A, Campitelli LF, Das PK, Yin Y, Albu M, et al. The Human Transcription Factors. Cell. 2018;172: 650–665. doi:10.1016/j.cell.2018.01.029

27. Gyorffy B, Surowiak P, Budczies J, Lánczky A. Online survival analysis software to assess the prognostic value of biomarkers using transcriptomic data in non-small-cell lung cancer. PLoS One. 2013;8. doi:10.1371/journal.pone.0082241

28. Mi H, Muruganujan A, Ebert D, Huang X, Thomas PD. PANTHER version 14: More genomes, a new PANTHER GO-slim and improvements in enrichment analysis tools. Nucleic Acids Res. 2019;47: D419–D426. doi:10.1093/nar/gky1038
